# High‐Pressure Reaction Profiles and Activation Volumes of 1,3‐Cyclohexadiene Dimerizations Computed by the Extreme Pressure‐Polarizable Continuum Model (XP‐PCM)

**DOI:** 10.1002/chem.202200246

**Published:** 2022-04-08

**Authors:** Bo Chen, K. N. Houk, Roberto Cammi

**Affiliations:** ^1^ Donostia International Physics Center Paseo Manuel de Lardizabal, 4 20018 Donostia-San Sebastián Spain; ^2^ IKERBASQUE Basque Foundation for Science Plaza Euskadi 5 48009 Bilbao Spain; ^3^ Department of Chemistry and Biochemistry University of California 90095 Los Angeles California USA; ^4^ Department of Chemical Science Life Science and Environmental Sustainability University of Parma Viale Parco Area delle Scienze. 17/a 43100 Parma Italy

**Keywords:** computational chemistry, cycloaddition, ene reaction, high-pressure chemistry, XP-PCM

## Abstract

Quantum chemical calculations are reported for the thermal dimerizations of 1,3‐cyclohexadiene at 1 atm and high pressures up to the GPa range. Computed activation enthalpies of plausible dimerization pathways at 1 atm agree well with the experiment activation energies and the values from previous calculations. High‐pressure reaction profiles, computed by the recently developed extreme pressure‐polarizable continuum model (XP‐PCM), show that the reduction of reaction barrier is more profound in concerted reactions than in stepwise reactions, which is rationalized on the basis of the volume profiles of different mechanisms. A clear shift of the transition state towards the reactant under pressure is revealed for the [6+4]‐ene reaction by the calculations. The computed activation volumes by XP‐PCM agree excellently with the experimental values, confirming the existence of competing mechanisms in the thermal dimerization of 1,3‐cyclohexadiene.

## Graphical abstract

**XP‐PCM calculations** on the thermal dimerizations of 1,3‐cyclohexadiene show that, going from the stepwise to the concerted [4+2] cycloaddition and finally to the concerted [6+4]‐ene mechanisms, as the facial overlap between the rings increases in the transition state structure, the volume of the transition state decreases, leading to a more negative activation volume and increasing reaction acceleration under pressure.

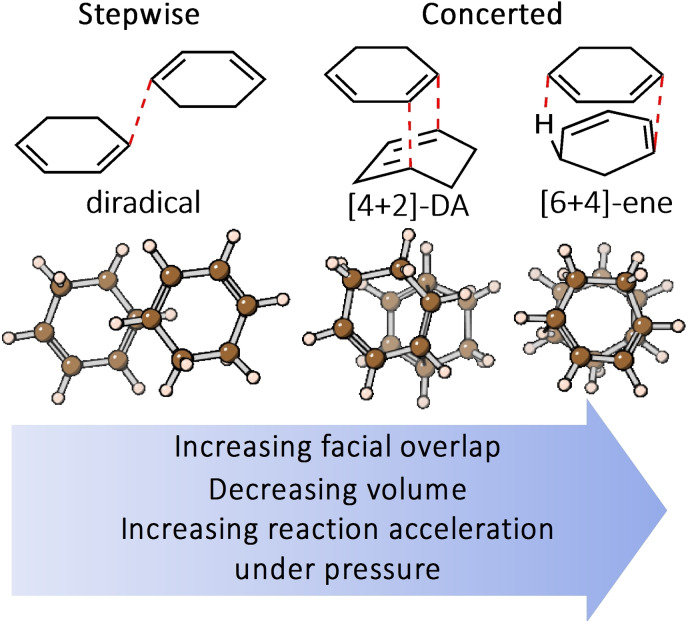

## Introduction

The rates of chemical reactions are usually affected by both temperature and pressure. The effect of temperature on the rate of a chemical reaction is measured as the activation energy.[^1^, ^2^] For thermally activated reactions (no tunneling^[3]^ involved), the larger the activation energy $E_{a}$ , the greater the increase in the reaction rate with temperature.^[4]^ Activation energies are measured experimentally by treating the temperature dependence of the rate constant using the Arrhenius equation. The activation energy is the slope of the plot of lnk vs. 1/T , $E_{a}=-R{\left(\frac{\partial lnk}{\partial (1/T)}\right)}_{p}$ , where k is the rate constant, T the temperature, R the ideal gas constant, and p the pressure.

The effect of pressure on the rate of a chemical reaction is measured by the activation volume (or volume of activation) ${\Delta V}^{\neq }$ .[^5^, ^6^, ^7^, ^8^, ^9^, ^10^, ^11^, ^12^, ^13^, ^14^, ^15^, ^16^, ^17^, ^18^] The activation volume is the volume change of a reaction system from the reactant(s) to the transition state. Experimentally, activation volumes are obtained by studying the pressure dependence of the rate constant using ${\Delta V}^{\neq }={\left(\frac{\partial {\Delta G}^{\neq }}{\partial p}\right)}_{T}=-RT{\left(\frac{\partial lnk}{\partial p}\right)}_{T},$ where $\Delta G^{\neq }$ is the Gibbs energy of activation, according to the thermodynamic formulation of the transition state theory.[^4^, ^19^, ^20^, ^21^]

**Figure 1:**
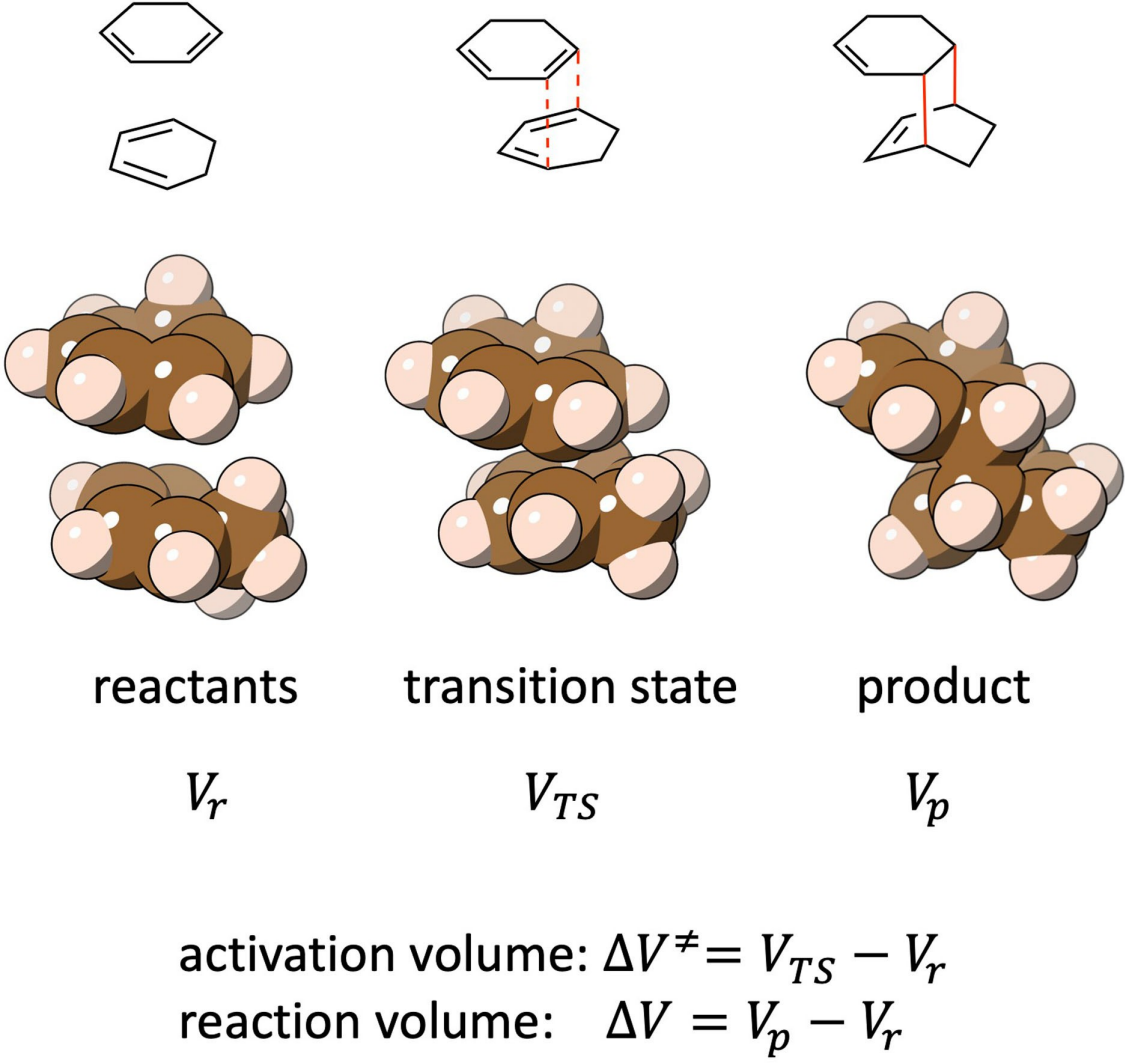
Schematic illustration of the activation volume and reaction volume (both are negative) of a Diels‐Alder dimerization of 1,3‐cyclohexadiene.

While activation energies are often positive,[^22^, ^23^, ^24^] activation volumes can commonly be both positive and negative. Reactions with negative and positive ${\Delta V}^{\neq }$ are accelerated and decelerated with increasing pressure, respectively. Usually, the signs of activation volumes can be intuitively predicted. For example, in the case of a bond‐forming reaction between two molecules (Figure 1), a negative ${\Delta V}^{\neq }$ is expected since the unimolecular transition state is more compact and hence has a smaller volume than the two separated reactant molecules. The opposite is usually true for bond cleavage reactions.

Measurements of ${\Delta V}^{\neq }$ are a valuable tool to distinguish different reaction mechanisms. In addition to $E_{a}$ , ${\Delta V}^{\neq }$ also provides information about the transition state (TS), arguably, in a more tangible manner, for the volume/size of a TS structure is directly related to its geometry. While competing mechanisms of a reaction can sometimes be difficult to distinguish based on $E_{a}$ , they may be distinguishable by ${\Delta V}^{\neq }$ when the competing TS structures have very different geometries. This is especially useful in studying the stepwise vs. concerted mechanisms of cycloadditions, given that the stepwise mechanism often has a less negative activation volume.[^25^, ^26^]

**Figure 2:**
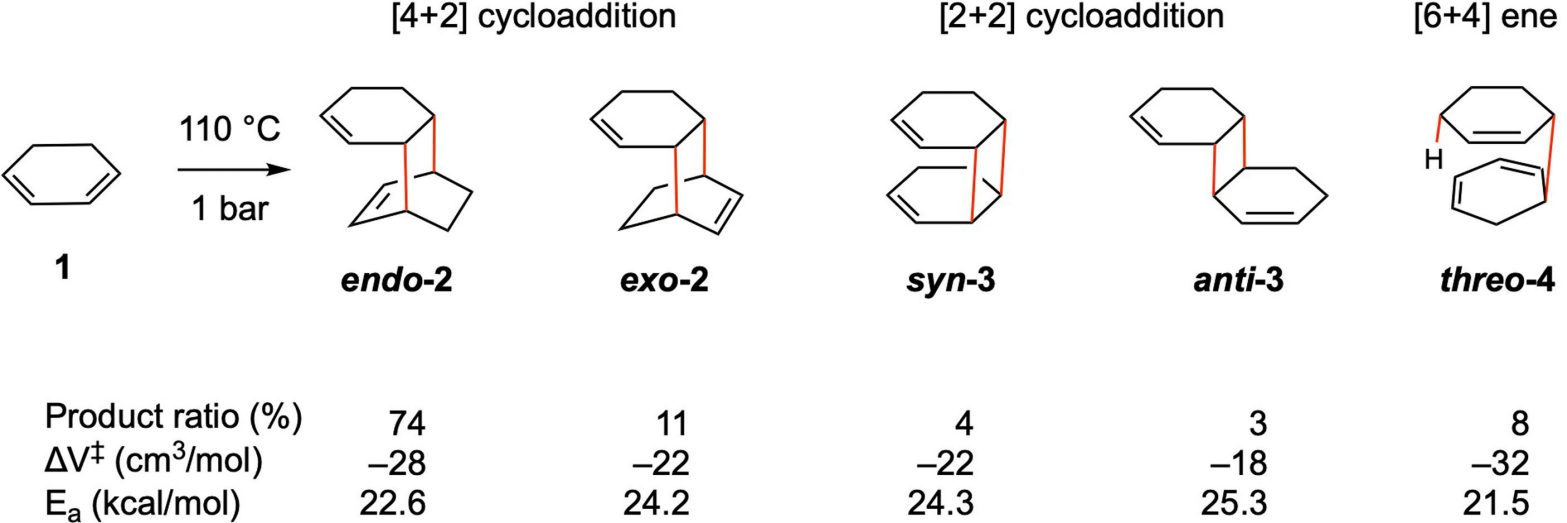
The observed products of thermal dimerizations of 1,3‐cyclohexadiene. New bonds are in red. Product ratio, experimentally measured activation volumes (${\Delta V}^{\neq }$ ) and activation energies ($E_{a}$ ) are shown.

In 1986, Klärner et al. reported that, at 1 atm, thermal dimerization of 1,3‐cyclohexadiene yielded five reaction products – two [4+2] cycloadducts of the *endo* and *exo* configurations, two [2+2] cycloadducts of the *syn* and *anti* configurations, and one untypical [6+4]‐ene adduct (Figure 2).^[27]^ Under pressure up to 7 kbar (about 0.7 GPa), the reactions yielding all five products are accelerated, indicating negative activation volumes for all these reactions. Considering the bond‐forming nature of these reactions, the observed acceleration under pressure is not surprising.

**Figure 3:**
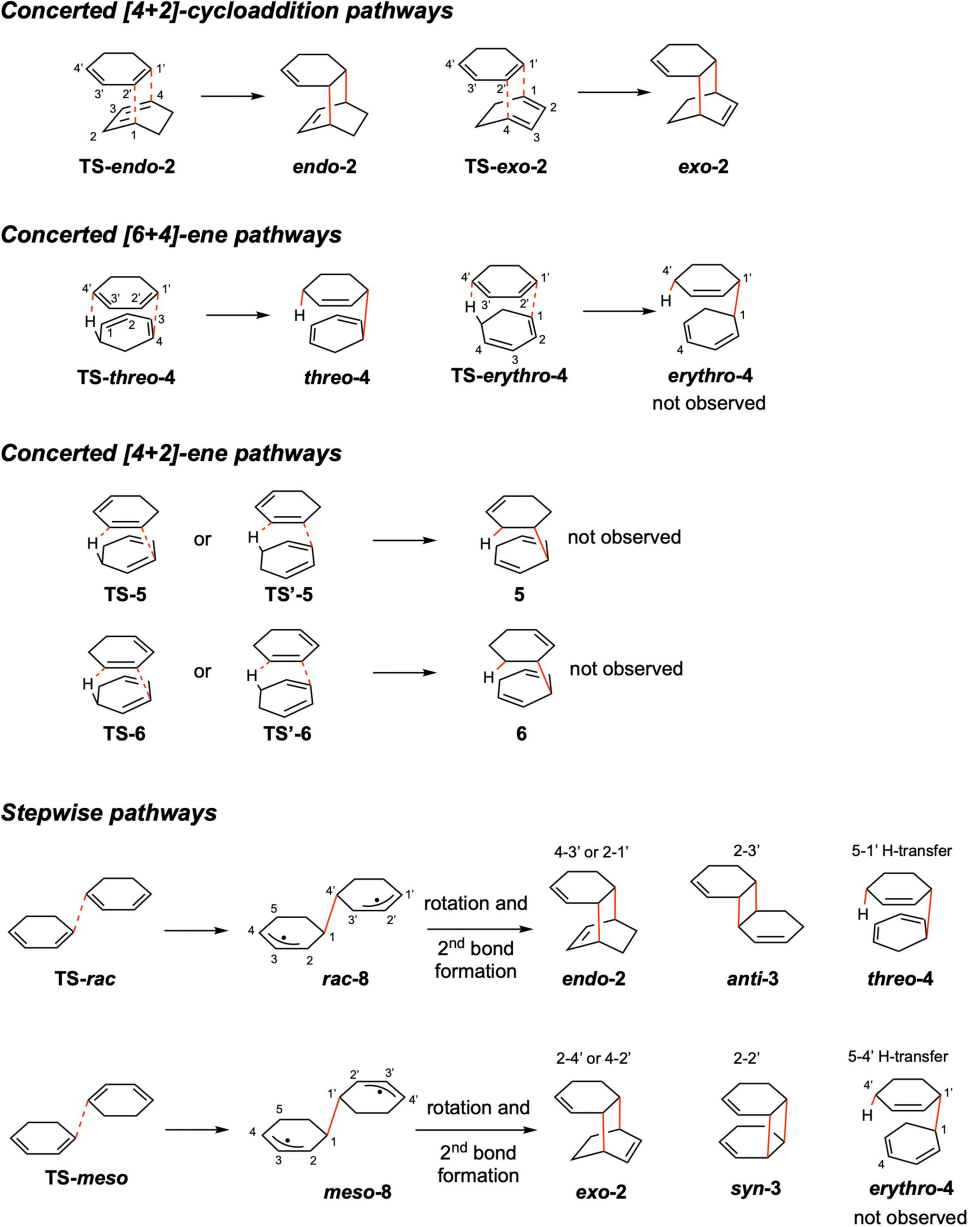
Plausible concerted and stepwise mechanisms in the dimerization of 1,3‐cyclohexadiene. The ring position numbering follows a clockwise fashion, with unprimed and primed numbers for the bottom and top rings, respectively.

Apparently, different mechanisms are in operation in this dimerization, for the adducts of both the symmetry‐allowed [4+2]‐cycloadditions and [6+4]‐ene reaction, and symmetry‐forbidden [2+2]‐cycloadditions, according to the Woodward‐Hoffmann rules,^[28]^ were observed. The adducts from symmetry‐allowed reactions could, in principle, be formed through either a concerted or stepwise mechanism, whereas the those from symmetry‐forbidden [2+2] cycloadditions are expected to be formed only via a stepwise mechanism under thermal conditions (Figure 3).[^27^, ^29^] The main difference between the concerted and stepwise mechanisms, in terms of activation volume, is that two new bonds are formed at once in a concerted mechanism and only one bond is initially formed in a stepwise mechanism, so that the concerted TS structure is usually more compact than the stepwise TS structure. Therefore, a concerted reaction typically has a more negative activation volume than a stepwise reaction.

Klärner et al. observed (Figure 2) that the reactions yielding the [4+2]‐cycloadduct ***endo*** **‐2** and the [6+4]‐ene adduct ***threo*** **‐4** were accelerated the most under pressure, with measured ${\Delta V}^{\neq }$ of −28 cm^3^/mol for the former and −32 cm^3^/mol for the latter.^[27]^ However, the reason why the [6+4]‐ene reaction has the most negative ${\Delta V}^{\neq }$ was not clear. Smaller ${\Delta V}^{\neq }$ between −22 and −18 cm^3^/mol were measured for the rest three reactions. Based on these ${\Delta V}^{\neq }$ data, it was reasoned that ***endo*** **‐2** and ***threo*** **‐4** are most likely formed by concerted mechanisms because their relatively large and negative ${\Delta V}^{\neq }$ suggest highly compact TS structures, whereas the other reactions with less negative ${\Delta V}^{\neq }$ are more compatible with stepwise mechanisms.^[27]^ Interestingly, the reactions affording the [4+2]‐adduct ***exo*** **‐2** and [2+2]‐adduct ***syn*** **‐3** were measured to have essentially the same activation volume and activation energy, which strongly indicates a common stepwise mechanism for these two very different reactions.^[27]^

Previous B3LYP, CASPT2 and CBS‐QB3 calculations by one of us and others show that the computed reaction barriers for the concerted [4+2]‐cycloadditions, concerted [6+4]‐ene reactions, and the stepwise additions (Figure 3) are within 5 kcal/mol.^[29]^ This small energy difference is consistent with the small difference in experimental $E_{a}$ , confirming the competitive nature of the concerted and stepwise mechanisms in the thermal dimerization of 1,3‐cyclohexadiene. However, the exact energetic order of these TS structures is slightly different between the calculation and experiment. In addition, activation volumes were not computed in this work.

Theoretical calculations of ${\Delta V}^{\neq }$ , especially from first‐principles, have been a challenge. A simple and intuitive way to compute ${\Delta V}^{\neq }$ is to calculate the difference in van der Waals (vdW) volumes of the molecule at the reactant and transition states. The vdW volume is the volume of interlocking vdW spheres (often with scaled vdW radii) centered on the constituting atoms of the molecule. However, likely due to intermolecular interactions and solvent effect being neglected, ${\Delta V}^{\neq }$ computed by this method are always too small in magnitude. An empirical packing coefficient was introduced to account for such negligence and to correct the underestimated ${\Delta V}^{\neq }$ , computed by this method.[^15^, ^30^]

A new method for more rigorous ${\Delta V}^{\neq }$ calculations is the recently‐developed extreme pressure polarizable continuum model (XP‐PCM).^[31]^ As an extension of the popular polarizable continuum model (PCM) that tackles molecular solvation energies under the standard condition of pressure, the XP‐PCM allows for quantum chemical calculations of the energy profiles of chemical reactions under high pressure. The effect of the pressure is introduced in XP‐PCM via a repulsive interaction between the molecular system and the surrounding solvent medium. Within XP‐PCM, ${\Delta V}^{\neq }$ can be computed, according to the transition state theory, as the derivative of activation free energy with respect to pressure. The XP‐PCM method has been applied to the calculations of the energy profiles of a subset of pericyclic reactions.[^17^, ^32^] Interesting phenomena such as a shift of the transition state and a switch of the rate determining step have been discovered by the calculations.^[17]^ Furthermore, the computed ${\Delta V}^{\neq }$ are in reasonable agreement with experimental values.^[17]^ A recent work by Fukuda and Nakatani applied the XP‐PCM method to a retrocycloaddition.^[33]^ The necessary details of the physical basis and computational protocol of the XP‐PCM method are given in the Computational Methodology section. In additional to reactions, the XP‐PCM method has also been applied to the studies of the effect of pressure on a variety of molecular properties, such as equilibrium geometries,[^34^, ^35^, ^36^] vibrational frequencies,[^37^, ^38^, ^39^] electronic excitation energies.^[40]^

We note here relevant approaches derived from the mechanochemistry field for high‐pressure calculations on molecules and reactions.[^41^, ^42^, ^43^, ^44^] Notably, the recent GOSTSHYP method from the Stauch group is capable of calculating activation volumes of reactions.^[45]^ Another approach employs a simulation box with periodic boundary condition, filled with solvated reactant molecules. The solvent molecules are explicitly included in the simulation box, in contrast to the implicit solvation approach in the XP‐PCM method. Molecular dynamics (MD) simulations or Monte Carlo simulations (an early example by Klärner et al.^[26]^) are performed to obtain reaction profiles at high pressures and activation volumes. Due to the large size of the system using a simulation box, the MD simulations were usually done with force fields, as illustrated in the works from the Weinberg group,[^46^, ^47^, ^48^, ^49^] or by a hybrid quantum mechanics/molecular mechanics (QM/MM) approach, as shown in the work by Plotnikov and Martinez^[50]^ and a recent work by Loco et al.^[51]^

We now report a thorough consideration of the potential energy surface (PES) of the thermal dimerization 1,3‐cyclohexadiene, including [4+2]‐ene pathways that were not considered previously. In addition, we report, for the first time, XP‐PCM calculations on the activation volumes for the various dimerization reactions of 1,3‐cyclohexadiene, offering a new approach for computationally studying the competing mechanisms of this reaction.

## Computational Methods

**Gas‐phase calculations**: Gas‐phase geometries were optimized at the ωB97XD^[52]^/def2‐TZVP^[53]^ level of theory. Frequency analyses were performed at the same level to verify the optimized structure to be either a minimum or a transition state, and to obtain the zero‐point vibrational, thermal, and entropic corrections, necessary in calculating enthalpies and free energies. For open‐shell singlets that appear in the stepwise mechanism, broken‐symmetry DFT with spin‐projection by the Yamaguchi‐Houk procedure^[54]^ was used. On the ωB97XD/def2‐TZVP optimized structures, single‐point calculations were performed using the strongly contracted N‐electron valence state perturbation theory (SC‐NEVPT2)[^55^, ^56^, ^57^] and coupled‐cluster CCSD(T)[^58^, ^59^] methods with the same def2‐TZVP basis set. In the SC‐NEVPT2 calculations, a (4,4) active space was used for 1,3‐cyclohexadiene, which comprises the valence π‐type orbitals and the valence π electrons in the molecules. An (8,8) active space was used for the transition states, which consists of the same set of π‐type orbitals and π electrons of two molecules of 1,3‐cyclohexadiene; some of these π‐type orbitals become more of σ‐type orbitals in the bond‐forming regions in the transition states. The ωB97XD and CCSD(T) calculations were performed using the Gaussian 16^[60]^ program package. The SC‐NEVPT2 calculations were done with the ORCA 4.2.1[^61^, ^62^] program package.

**Figure 4:**
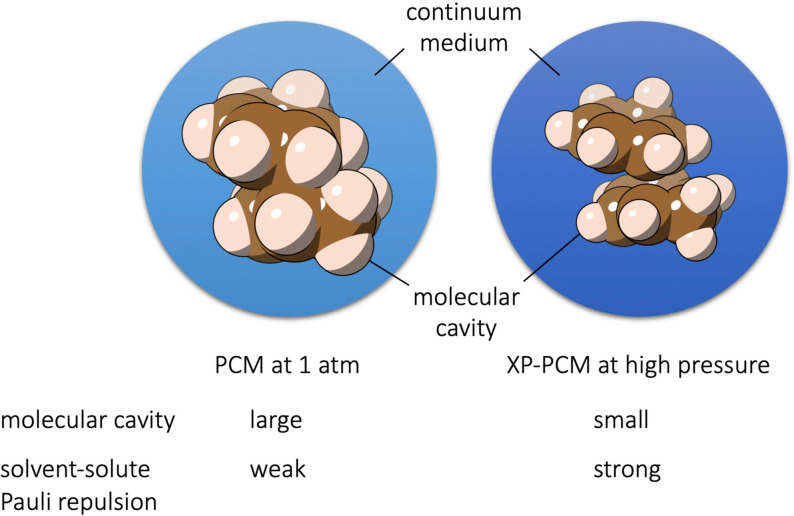
The XP‐PCM model as an extension of the PCM model. A molecular system is confined in a molecular‐shaped cavity (in this case the vdW cavity) in the external medium (in blue). The combination of a smaller size of the cavity and a stronger solvent‐solute Pauli repulsion in XP‐PCM models the effect of compression on the molecule.

**High‐pressure calculations**: High pressure calculations (up to a few GPa) were performed using the XP‐PCM method at the ωB97XD/def2‐ΤZVP level of theory with Gaussian 16 and an in‐house script. The XP‐PCM is a quantum chemical method aimed to introduce the effects of the pressure on the calculation of the electronic energy $G_{er}$ of a molecular system in a dense medium via a Pauli exchange‐repulsion interaction between the molecular system and the external medium. Such a Pauli‐exchange repulsion is motivated by the fact that at high pressure, the reduction of the volume of a dense medium forces the intermolecular distances below the van der Waals contacts, where the intermolecular interactions are dominated by the Pauli exchange‐repulsion.^[63]^ The increase of the pressure is modeled by simply shrinking the volume $V_{c}$ of the cavity hosting the molecular system so as to increase the overlap between the electron densities of the system and of the external medium (Figure 4). This molecular cavity is built up starting from the envelope of vdW spheres centered on the nuclei of the molecular system and with scaled vdW radii. In the actual calculation, a cavity enclosed in the solvent‐excluded surface (SES)^[64]^ is used, and we call this cavity the SES cavity.

In studying chemical reactions at high pressure, the effective potential energy for the motion of the nuclei of the reactive system $G_{tot}\left(p\right)$ is the sum of the electronic energy $G_{er}\left(p\right)$ and the so‐called cavitation Gibbs energy $G_{cav}\left(p\right)$ that corresponds to the work necessary in order to create the void cavity at the given condition of pressure p :

(1) $$G_{tot}\left(p\right)=G_{er}\left(p\right)+G_{cav}\left(p\right)$$

According to the scaled particle theory, the cavitation energy $G_{cav}\left(p\right)$ consists of volume work $pV_{c}$ (pressure times the cavity volume) and an entropic contribution related to the numeric density (which depends on the pressure) of the solvent $G_{non-pV}\left(p\right)$ :^[65]^

(2) $$G_{cav}\left(p\right)=pV_{c}+G_{non-pV}\left(p\right)$$

The use of the symbol G is consistent with previous works of the XP‐PCM method[^17^, ^31^] and the traditional PCM method.^[66]^ Note that $G_{tot}\left(p\right)\;$ does not contain zero‐point vibrational corrections, thermal corrections or entropic correction, assuming their negligible contributions to the reaction profile. The effective potential energy profile for a reaction at a given pressure p is determined by computing $G_{tot}\left(p\right)$ for a set of selected structures along a suitable gas‐phase reaction coordinate. Note that the geometries were not optimized at high pressure; we are in the process of implementing analytical gradient of the XP‐PCM total energy, which is needed for performing (transition state) geometry optimization. The effect of pressure on covalent bond distances is rather small, for instance, the C−C and C−H distances in benzene shorten no larger than 0.02 Å going from 1 atm to 20 GPa.^[67]^ The reaction barrier is then computed as the difference in $G_{tot}\left(p\right)$ between the TS and reactant(s), ${\Delta G}_{tot}^{\neq }\left(p\right)$ . According to the transition state theory, the activation volume ${\Delta V}^{\neq }$ is determined from the slope of the corresponding reaction barrier ${\Delta G}_{tot}^{\neq }\left(p\right)$ as a function of the pressure,

(3) $${\Delta V}^{\neq }=d{\Delta G}_{tot}^{\neq }\left(p\right)/dp$$

Detailed XP‐PCM parameters are given in the Supporting Information (Supporting Information). We recommend that interested readers consult ref. [17] for a detailed tutorial about the protocol. All XP‐PCM calculations are performed for selected structures (reactants, transition state, product) along the gas phase intrinsic reaction coordinates.

### Potential energy surface at 1 atm

As shown in Figure 3, the concerted pathways considered in this paper include [4+2]‐cycloadditions, [6+4]‐ene reactions, and [4+2]‐ene reactions. The [4+2]‐ene pathways were not considered in previous calculations by Ess et al.^[29]^ For the concerted [4+2]‐cycloadditions and [6+4]‐ene reactions, each of these pathways can generate two stereo isomers as a result of different relative orientations of the two cyclohexadiene rings in the TS structures. These isomers are termed *endo* and *exo* for the [4+2] cycloaddition adducts and *threo* (also could be called *rac* or *dl*) and *erythro* (also could be called *meso*) for the [6+4] ene‐adducts.

For concerted [4+2]‐ene reactions, two pathways are possible. In each of them, there exist configurational isomers for the TS, for example, **TS‐5** and **TS’‐5**. However, both of these two TS structures lead to the same dimer (i. e., **5**). The configurational isomerism in the TS disappears in the dimer because the bottom ring in the adduct becomes 1,4‐cyclohexadiene that does not contain any stereocenter.

For stepwise pathways, we considered those beginning with a C−C bond formation between two 1,3‐cyclohexadiene molecules at the terminus of the diene moieties and generating a diallyl intermediate, ***rac*** **‐8** or ***meso*** **‐8**. Either of the two intermediates contains two stereocenters, but the ***meso*** **‐8** isomer is achiral due to the presence of an inversion center in the structure. Other stepwise pathways involving bond formation at internal sites of the diene moiety are likely to be unfavorable due to the generation of isolated (i. e., not in conjugation with a double bond) radical sites; these pathways are not considered. In the second step, a rotation about the first formed C−C bond followed by a radical recombination or an H‐transfer leads to a cycloaddition or ene product. For example, a clockwise rotation of the top ring in ***rac*** **‐8** intermediate ∼60 degrees about C1−C4’ followed by a radical recombination at C2 and C3’ generates ***anti*** **‐3**. A rotation of the same ring ∼120 degrees followed by the C4−C3’ radical recombination yields ***endo*** **‐2**. A further clockwise rotation (∼150 degrees) bringing C5 and C1’ in proximity could enable H‐transfer from C5 to C1’, leading to the ene product ***threo*** **‐4**. Similar analysis for ***meso*** **‐8** shows that ***exo*** **‐2**, ***syn*** **‐3** and ***erythro*** **‐4** can be formed from it, though ***erythro*** **‐4** was not observed experimentally.

**Figure 5:**
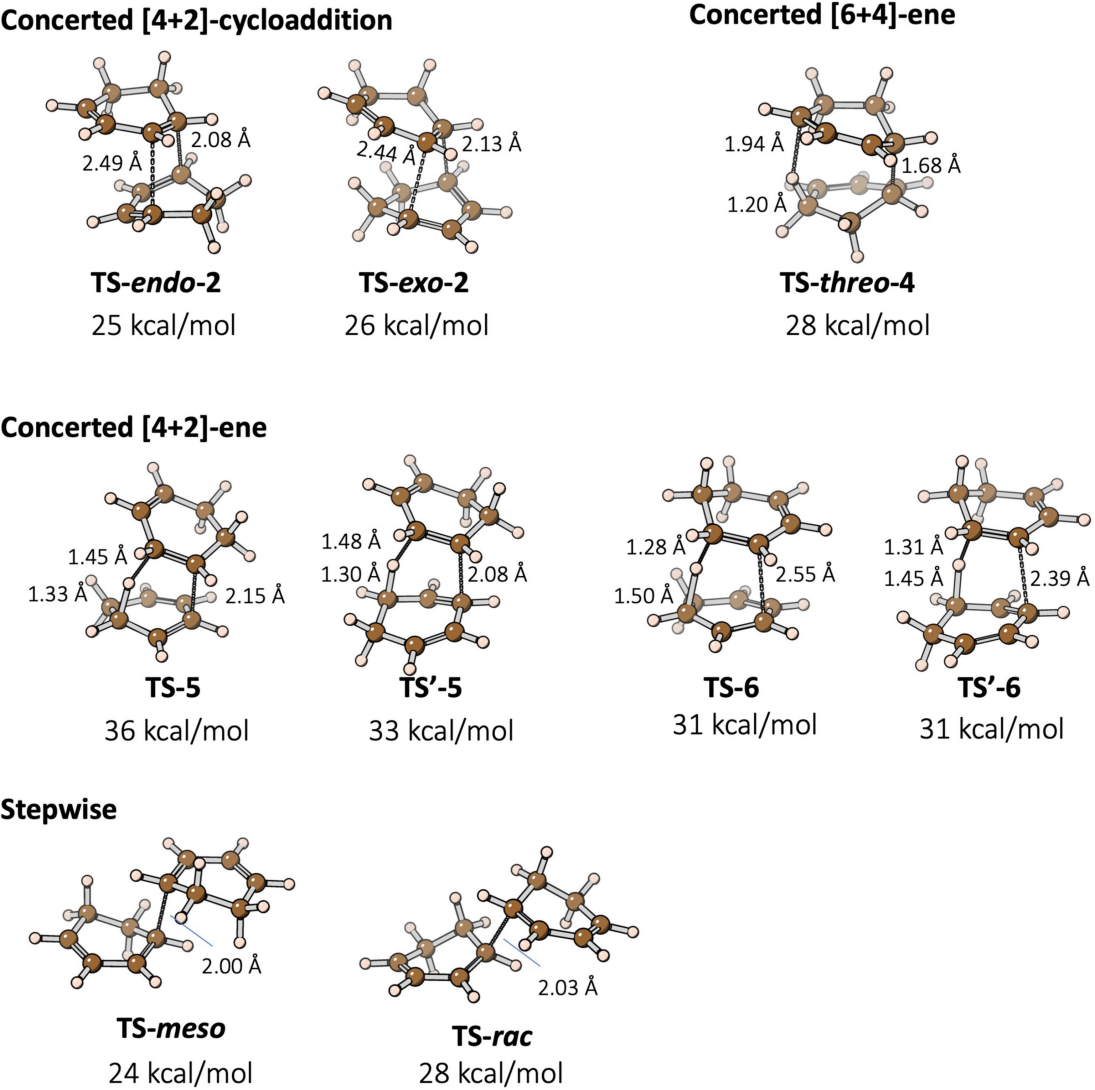
Structures and gas phase enthalpies $\Delta H_{298K}^{\neq }$ of the considered TS structures relative to two isolated molecules of 1,3‐cyclohexadiene, computed at the ωB97XD/def2‐TZVP level of theory. Key bond distances are shown.

A thorough discussion on the 1 atm potential energy surface of this complicated dimerization reaction is given in the Supporting Information. Here we present the most important features of these 1 atm reaction profiles, which are the basis for later discussions on the high‐pressure reaction profiles. The ωB97XD/def2‐TZVP computed TS structures are shown in Figure 5. At this level of theory, the *endo* [4+2] cycloaddition pathway going through **TS‐*endo*‐2** is calculated to have the lowest enthalpic barrier of 25 kcal/mol. The barrier of the *exo* pathway is 1 kcal/mol higher, likely due to the absence of secondary orbital interactions^[68]^ in **TS‐*exo*‐2**. Distortion‐interaction analysis^[69]^ (see Supporting Information) on the TS structures show that **TS‐*endo*‐2** has the smallest distortion energy and a reasonable interaction energy, leading to the smallest electronic energy barrier of this pathway.

ωB97XD/def2‐TZVP calculations also show that the activation enthalpy of the *threo* [6+4]‐ene reaction going through **TS‐*threo*‐4** is 3 kcal/mol higher than that of the *endo* [4+2] cycloaddition. However, CCSD(T)/def2‐TZVP single‐point calculations give opposite results that the *threo* [6+4]‐ene pathway is the most favored pathway, with **TS‐*threo*‐4** being 1 kcal/mol lower than **TS‐*endo*‐2**. The search for the TS structure of a second concerted [6+4]‐ene pathway, **TS‐*erytho*‐4**, was not successfully and always led to either the [4+2] cycloaddition TS structure **TS‐*exo*‐2** or the [4+2]‐ene TS structure **TS‐6**. We show in the Supporting Information that these three TSs have similar structures. One might have even anticipated the formation of an ambimodal transition state[^70^, ^71^] leading to several of these products. These calculations are consistent with the [6+4]‐ene adduct ***erytho*** **‐4** not being observed experimentally.

The computed enthalpic barriers of [4+2]‐ene reactions are 2–11 kcal/mol higher than those of the [4+2] cycloaddition and [6+4]‐ene pathways. A plausible reason for the higher energies of the [4+2]‐ene TS structures is that the conjugation between the dienes in the bottom ring (Figure 3) is lost in the [4+2]‐ene TS structures whereas it remains in the TS structures of the [4+2]‐cycloadditions and [6+4]‐ene reaction. These >30 kcal/mol large barriers of the [4+2]‐ene reactions are consistent with the fact that [4+2]‐ene products are not observed experimentally.

For the stepwise pathways, the first C−C forming TS is computed to be the rate‐determining TS. At the ωB97XD/def2‐TZVP level, both the spin‐contaminated or the spin‐projected energies shows that the stepwise **TS‐*meso*** is lower in energy than the concerted **TS‐*endo*‐2**, in contradiction to the experimental activation energies $E_{a}$ in Figure 2. However, at the NEVPT2(8,8)/def2‐TZVP//(U)ωB97XD/def2‐TZVP level, **TS‐*meso*** was computed to be 1 kcal/mol higher in enthalpy than **TS‐*endo*‐2**, in agreement with the experimental $E_{a}$ . **TS‐*rac*** is computed to be 3–5 kcal/mol higher in energy than **TS‐*meso*** at the ωB97XD and NEVPT2 levels, consistent with previous calculations;^[29]^ however, this difference is only 1 kcal/mol in experimental $E_{a}.$

### Reaction profiles under pressure

**Figure 6:**
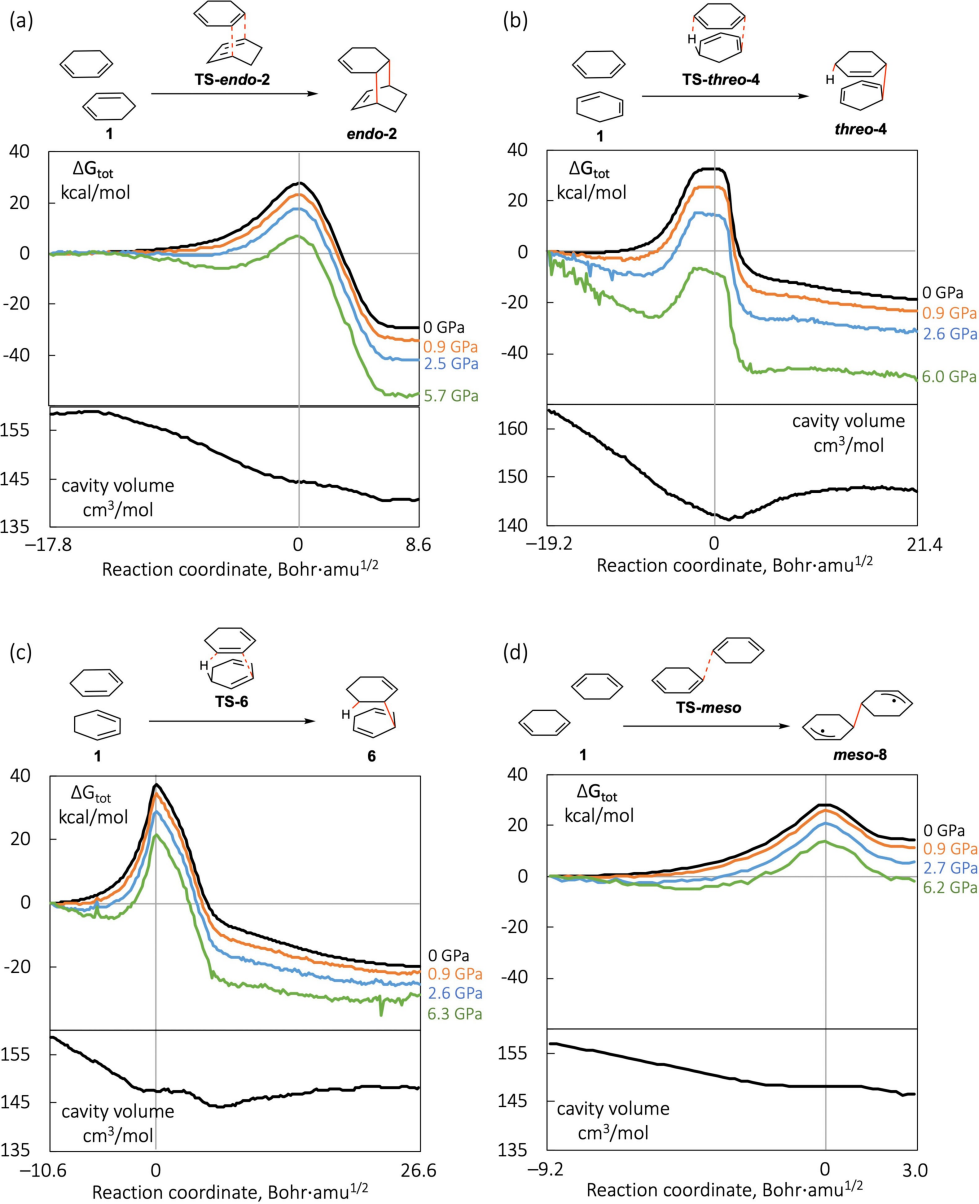
Effective reaction profiles ($\Delta G_{tot}$ in Equation (1)) at different pressures and cavity volume profiles (SES cavity with a scaling factor of 1.2 of the Bondi radii) of concerted [4+2] cycloaddition, concerted [6+4]‐ene reaction, and the first step of the *meso* stepwise addition of 1,3‐cyclohexadiene dimerization, calculated by the XP‐PCM method at the ωB97XD/def2‐TZVP level. The 0 GPa curves are gas‐phase intrinsic reaction coordinates (IRC) calculations with >100 points; the high‐pressure curves are single‐point XP‐PCM calculations based on the gas‐phase IRC.

Figure 6 shows the computed profiles of the effective energy $\Delta G_{tot}$ (see Equation (1)) and cavity volume profiles of the four types of dimerization pathways in Figure 3 or Figure 5. For each type of reaction, one stereo version is shown and the others are given in the Supporting Information. For example, only the *endo* [4+2] cycloaddition is shown in Figure 6 and the *exo* cycloaddition is given in the Supporting Information. The profiles at 0 GPa (gas phase) are from the intrinsic reaction coordinate calculations; the high‐pressure profiles are computed using the XP‐PCM method based on the 0 GPa structures.

A first look shows that the four types of reaction have very different profiles, for example, in the location of the TS structure along the reaction coordinate and in the shape of the $\Delta G_{tot}$ and volume profiles. They do share a common feature – the $\Delta G_{tot}$ profile decreases in energy as the pressure increases, which is consistent with the expected negative activation volumes of these dimerizations.

**Concerted [4+2] cycloaddition**: The concerted [4+2] cycloaddition has a fairly late TS at about 2/3 of the entire reaction course. This reaction profile is similar to other [4+2] cycloadditions previously studied using the same XP‐PCM method.[^17^, ^72^, ^73^] In the current *endo*‐cycloaddition, the first 1/4 of the PES is rather flat, where two isolated 1,3‐cyclohexadiene molecules reorient themselves to achieve a proper geometry for the cycloaddition and then begin to approach one another; the latter motion would lead to a decrease of the cavity volume. At high pressure, a minimum of a vdW complex develops prior to the TS, as noted previously by some of us[^17^, ^72^] and by Loco et al.^[51]^ The minimum is very shallow at low pressures (<1 kcal/mol below the structure at reaction coordinate=0) but becomes apparent at 5.7 GPa. This minimum also shifts towards the TS as the pressure increases. The emergence of a pre‐TS minimum and the shift of it towards the TS could be explained by the pressure‐enhanced vdW complex formation. The higher the pressure, the shorter the vdW separation between the molecules, and the smaller the volume of such complex. Then, the favorable pV term of the enthalpy leads to the appearance of a minimum for the complex. Another explanation for the pre‐TS minimum is provided by the 2^nd^‐order effect of the mechanical force on the initial part of the PES.^[74]^ This phenomenon of a pre‐TS minimum seems to be common in bimolecular reactions; the other three reactions in Figure 6 also exhibits such a feature, especially in the concerted [6+4]‐ene reaction, where deep pre‐TS minima at high pressures are revealed by the calculations.

The cavity volume profile of the *endo* [4+2] cycloaddition also shows a flat region at the beginning of the reaction; this is where the two molecules reorient themselves with a rotation before approaching each other. Afterwards, the cavity volume, as defined in the PCM model, decreases monotonically during the course of bond formation and does not change much when the bond formation is complete at the end of the profile. A nice correspondence between the cavity volume profile and the $\Delta G_{tot}$ profile is evident – at the beginning and end of the reaction, the volume and $\Delta G_{tot}$ profiles are both flat, whereas during the bond formation, the decreasing volume is matched by the enthalpy decrease at high pressures.

**Figure 7:**
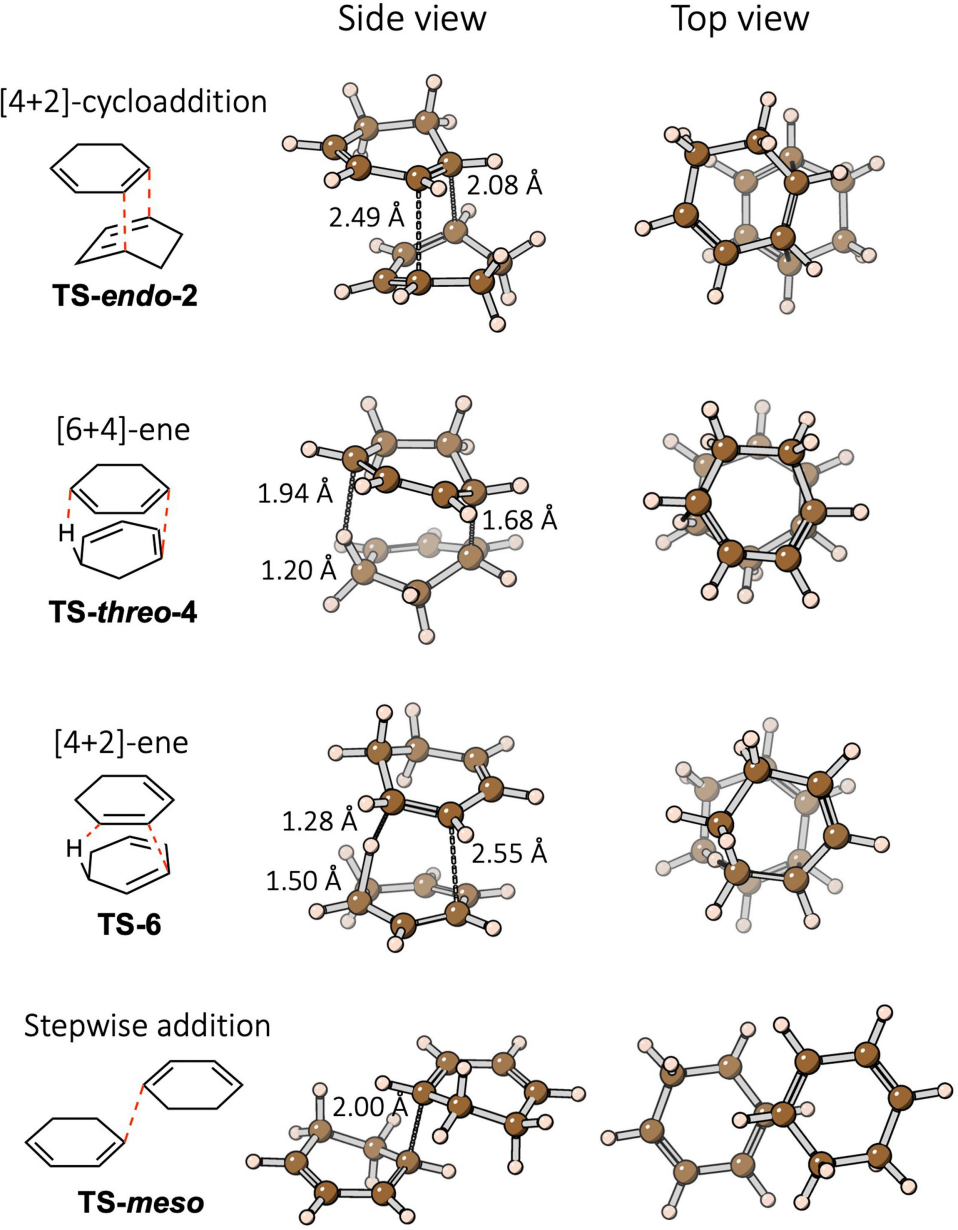
Two views of the ωB97XD/def2‐TZVP optimized gas‐phase TS structures with key bond distances shown.

**Concerted [6+4]‐ene reaction**: In the *threo* type [6+4]‐ene reaction (Figure 6b), the concurrent C−C bond formation and H‐transfer at the 1,4 positions of one ring and 1,5 positions of the other results in a substantial overlap of the two rings in the TS, more than that in the [4+2] cycloaddition TS. A top view (Figure 7) of this TS structure, **TS‐*threo*‐4**, shows that the two rings are almost coaxial and the CH/CH_2_ groups of the two rings are in a staggered conformation. In contrast, **TS‐*endo*‐2** has less overlap between the two rings as shown in the top view. The greater overlap of the rings in **TS‐*threo*‐4** leads to a more compact structure with a smaller volume, compared with **TS‐*endo*‐2**. The volume of the SES cavity of **TS‐*threo*‐4** is computed to be 142 cm^3^/mol, compared with 145 cm^3^/mol for **TS‐*endo*‐2**. Note that 1 cm^3^/mol difference in volume corresponds to 0.24 kcal/mol difference in enthalpy at 1 GPa. The greater overlap of the rings and the highly‐ordered staggered conformation in **TS‐*threo*‐4** is correlated with the small computed entropy of this TS and the small experimental pre‐exponential factor in the Arrhenius equation for this *threo* [6+4]‐ene reaction (see the discussion on the 1 atm PES in Supporting Information), compared with other reactions.

The 0 GPa (gas phase) PES of the *threo* [6+4]‐ene reaction has a rather flat region near the TS (Figure 6b), where the C⋅⋅⋅C and C⋅⋅⋅H distances of the forming bonds (indicated by red dashes in **TS‐*threo*‐4**) change from 1.9 Å and 2.3 Å to 1.7 Å and 1.8 Å, respectively. After this flat region of the PES, the H‐transfer completes in a few steps with a fast and substantial energy decrease. Then, a long and slow conformational change takes place in the last 1/3 of the reaction process. This conformation change mainly features the separation of the C, from which the H is transferred, from the newly formed C−H bond. Ignoring the last 1/3 of the profile of conformational change, this reaction also has a very late TS. The cavity volume of the system decreases from the beginning of the reaction to about the point at which the H‐transfer completes (i. e., a bit past the TS). Then the cavity volume increases, as the ene‐adduct opens up to adapt to its optimal conformation at 0 GPa.

At high pressures, a pre‐TS vdW complex minimum develops, similar to the case of the [4+2] cycloaddition. But this minimum in the [6+4]‐ene reaction is much deeper compared with that in the [4+2] cycloaddition. A most likely reason is that the separated reactant molecules occupy a larger volume (164 cm^3^/mol) than in the [4+2] cycloaddition (158 cm^3^/mol), so that a greater volume reduction is realized in forming the vdW complex. The flat TS region of the PES at 0 GPa becomes tilted with the post‐TS region lowering in enthalpy at high pressures, due to the monotonical volume decrease in this region. A clear shift of the TS towards the reactant is seen at high‐pressures. The shift of TS at high pressure is consistent with the Hammond postulate^[75]^ that the TS shifts towards the reactant when the reaction becomes more exothermic. However, note that the TS shift is apparent only in the [6+4]‐ene reaction, much less so in the other three reactions in Figure 6, though all these reactions become more exothermic (or less endothermic) at high pressures. One distinct feature of the [6+4]‐ene reaction is that the cavity volume keeps decreasing in the TS region, while in the other three reactions, the cavity volume stays almost constant in the TS region. Therefore, the effect of pressure is larger on the PES in the TS region in the [6+4]‐ene reaction, leading to an apparent shift of the TS towards the reactant. The shift of TS along reaction coordinate under pressure has been noted previousely.[^17^, ^41^, ^49^, ^50^]

Without doing geometry optimization of the transition state under pressure, it is demonstrated from the example above that the effect of pressure on the geometry of, in particular, transition state, can be studied by monitoring the shift of the maximum of the reaction profile under pressure.

**Concerted [4+2]‐ene reaction**: In contrast to the flat TS region in the [6+4]‐ene reaction, the [4+2]‐ene reaction has a sharp maximum in the TS region of the PES (Figure 6c). We inspected the TS structures in the two reactions to understand the reason. In the [6+4]‐ene **TS‐*threo*‐4**, the C−C bond is almost fully formed at a distance of 1.68 Å while the H is barely transferred with distances of 1.20 and 1.90 Å for the breaking and forming C−H bonds (Figure 7). On the contrary, in the [4+2]‐ene **TS‐6**, the H‐transfer is substantial with 1.50 and 1.28 Å for the two C−H bonds but the C−C bond to be formed is at a large distance of 2.55 Å. The shapes of PESs near the TSs and the structural features of the TSs in these two ene reactions suggest that the H‐transfer is associated with a much larger energy change than the C−C bond formation. When the TS structure of the ene reaction is dominated by C−C bond formation, the PES near the TS is flat, as in the [6+4]‐ene reaction; whereas when the TS structure is governed by H‐transfer, the PES near the TS is sharp, as in the [4+2]‐ene reaction.

The [4+2]‐ene reaction also has a long course of conformational change in the second half of the profile. Again, this is due to the opening up of the molecule after the H‐transfer. Correspondingly, the volume increases during the course of this conformational change (Figure 6c). Interestingly, the volume profile has a flat region near the TS; the reason is two‐fold. In the region before the TS, where H‐transfer starts to take place, the C⋅⋅⋅C distance in the C−H⋅⋅⋅C moiety stays almost constant (at about 2.8 Å), so does the distance of the C−C bond to be formed (at about 2.6 Å). During this course, there is little movement of the two molecules towards each other, which leads to little volume change. In the region after the TS, the C−C bond starts to form, which reduces the separation between the two rings and should lead to volume reduction. However, this effect is counter‐balanced by the opening‐up of the C⋅⋅⋅H−C moiety after H‐transfer that would result in a volume increase. The overall effect is that the volume stays constant in quite a region after the TS. Afterwards, the C−C bond formation dominates for a short duration with a decrease of the cavity volume, before the final conformational change takes over and leads to a slow volume increase.

The cavity volume of the [4+2]‐ene **TS‐6** is 147 cm^3^/mol, larger than those of the **TS‐*endo*‐2** in the [4+2]‐cycloaddition (145 cm^3^/mol) and **TS‐*threo*‐4** in the [6+4]‐ene reaction (142 cm^3^/mol). Consequently, the lowering of the enthalpy at high pressure is much less in the [4+2]‐ene TS than in the other two reactions. As discussed above, because the cavity volume does not change in the TS region in the [4+2]‐ene reaction, one would not expect a shift the TS location under pressure (Figure 6c).

**Stepwise addition**: Moving on to the stepwise mechanism, Figure 6d shows the reaction profiles of the *meso* addition. Compared with the above‐discussed concerted reactions, this *meso* addition has a rather short course, especially in the post‐TS region. The C−C distance of the forming bond changes from 2.00 Å in **TS‐*meso*** to 1.57 Å in the adduct ***meso*** **‐8**. The cavity volume of the system decreases in the *meso*‐addition, but with a much less volume reduction compared with the above concerted reactions where two bonds are formed. Consequently, an increase in pressure provides much less reduction of the reaction barrier for the *meso* addition. The *meso*‐addition to form the intermediate ***meso*** **‐8** is calculated to be enthalpically favorable only at pressures higher than about 6.2 GPa.

### Activation volumes

As shown in Table 1, the computed activation volume ${\Delta V}^{\neq }$ by Equation (3) are −27.7 and −28.2 cm^3^/mol for the concerted *endo* and *exo* [4+2] cycloadditions (see Supporting Information for the numerical calculations of activation volumes). Less negative ${\Delta V}^{\neq }$ of −20.5 and −21.4 cm^3^/mol are computed for the stepwise mechanisms that may also deliver the two [4+2] cycloadducts. Comparison with experimental ${\Delta V}^{\neq }$ highly suggests that the *endo* [4+2] cycloaddition follows a concerted mechanism, but the *exo* adduct is formed through a stepwise mechanism.

**Table 1:** Calculated (using Equation (3) at the ωB97XD/def2‐TZVP level of theory) and experimental activation volumes ${\Delta V}^{\neq }$ .

Reaction product	computed ${\Delta V}^{\neq }$ in cm^3^/mol	experimental ${\Delta V}^{\neq }$ in cm^3^/mol
***endo*** **‐2** [4+2]‐cycloadduct	−27.7 (concerted) −20.5 (stepwise via **TS‐*rac***)	−28
***exo*** **‐2** [4+2]‐cycloadduct	−28.2 (concerted) −21.4 (stepwise via **TS‐*meso***)	−22
***threo*** **‐4** [6+4]‐ene adduct	−32.1 (concerted)	−32
***syn*** **‐3** [2+2]‐cycloadduct	−21.4 (stepwise via **TS‐*meso***)	−22
***anti*** **‐3** [2+2]‐cycloadduct	−20.5 (stepwise via **TS‐*rac***)	−18

The concerted [6+4]‐ene reaction is computed to have the most negative ${\Delta V}^{\neq }$ of −32.1 cm^3^/mol, in excellent agreement with the experimental value, thus conforming the concerted mechanism of this reaction. This large negative ${\Delta V}^{\neq }$ for the [6+4]‐ene reaction is consistent with the compact geometry of the TS structures, as discussed in the section of reaction profile under pressure.

For the [2+2]‐cycloadduct ***syn*** **‐3**, the computed ${\Delta V}^{\neq }$ of the stepwise mechanism through the ***meso*** **‐8** intermediate is in excellent agreement with the corresponding experimental data. For ***anti*** **‐3**, the computed ${\Delta V}^{\neq }\;$ of the stepwise mechanism through the ***rac*** **‐8** intermediate is in good agreement with the experimental data with a slight overestimation. These calculations support stepwise mechanisms for the [2+2] cycloadditions.

The computed activation volume, ${\Delta V}^{\neq }$ , reflecting the kinetic effect of the pressure, originates from several contributions. In fact, from Equations 1–3, we obtain ${\Delta V}^{\neq }$ =${d\Delta G}_{tot}^{\neq }/dp$ =${d(\Delta G}_{er}^{\neq }+{\Delta G}_{cav}^{\neq })/dp$ =$d({\Delta G}_{er}^{\neq }+p{\Delta V}_{c}^{\neq }+{\Delta G}_{non-pV}^{\neq })/dp$ =${d\Delta G}_{er}^{\neq }/dp+{\Delta V}_{c}^{\neq }+d{\Delta G}_{non-pV}^{\neq }/dp$ .

**Figure 8:**
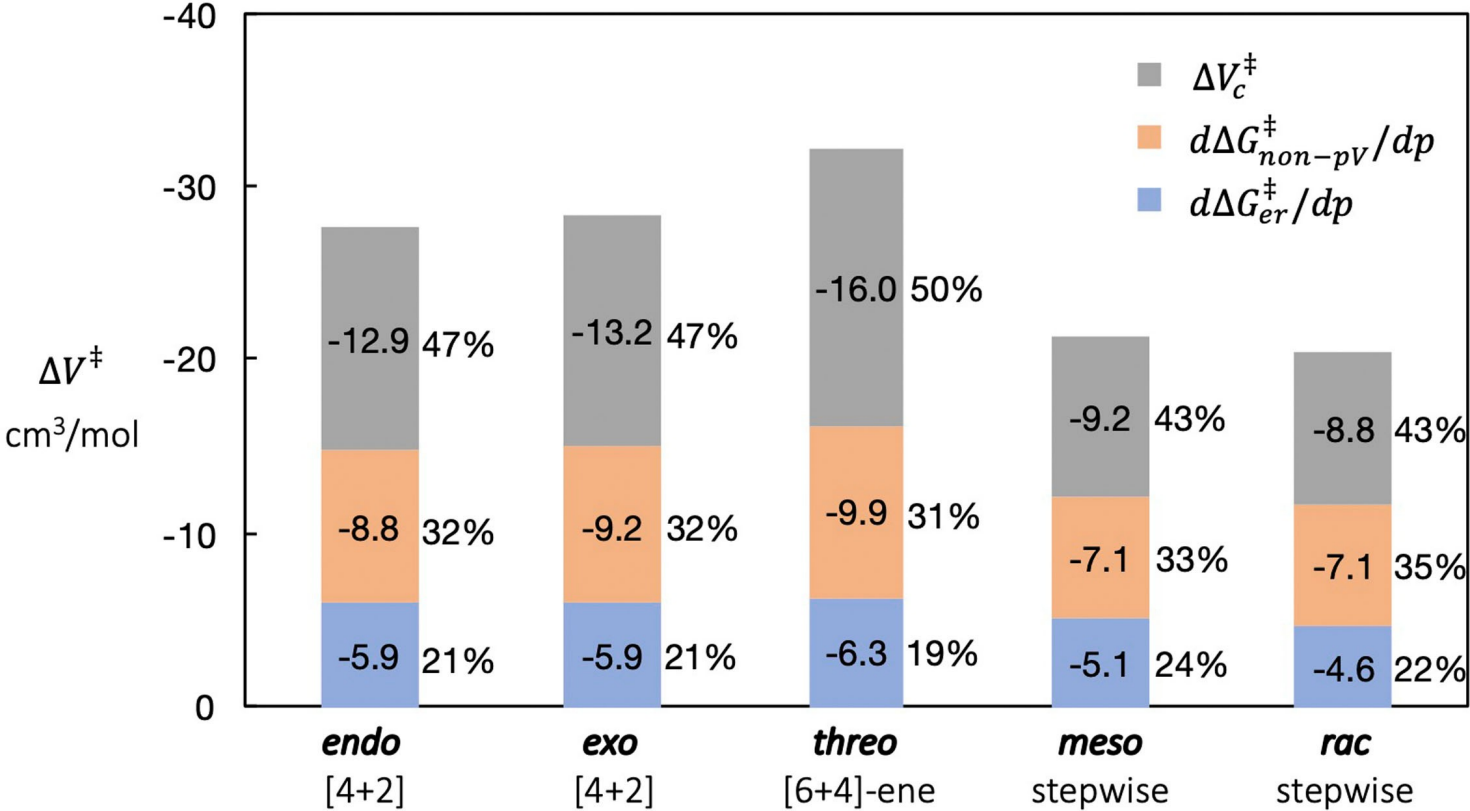
The partition of the computed ${\Delta V}^{\neq }$ into the effect on electronic energy ${\Delta G}_{er}^{\neq }$ , and the effect on the non‐pV terms of the cavitation energy ${\Delta G}_{non-pV}^{\neq }$ and the change of cavity volume ${\Delta V}_{c}^{\neq }$ .

This is to say that the computed ${\Delta V}^{\neq }$ can be partitioned into the contribution from the effect of pressure on the change of electronic energy ${\Delta G}_{er}^{\neq }$ , the change of the cavity volume ${\Delta V}_{c}^{\neq }$ , and the contribution from the effect of pressure on the change of the non‐pV term of the cavitation energy ${\Delta G}_{non-pV}^{\neq }$ . As shown in Figure 8, the change in cavity volume ${\Delta V}_{c}^{\neq }$ has the largest contribution to activation volume ${\Delta V}^{\neq }$ , but no larger than 50 %. This means that calculating activation volume ${\Delta V}^{\neq }$ as the change of cavity volume ${\Delta V}_{c}^{\neq }$ , as was done previously,[^15^, ^30^] would lead to significant underestimation.

Comparison of the percentage contributions of the three components to activation volume across the five reactions shows that, for the same type of reaction, such as *endo* vs. *exo* [4+2], or *meso* vs. *rac* stepwise addition, the percentage contributions are similar, likely due to the similar TS structure and cavity shape.

Why do all three components of ${\Delta V}^{\neq }$ in Figure 8 contribute a negative activation volume for all these five reactions? It is easy to understand that ${\Delta V}_{c}^{\neq }$ is negative because these reactions are bond‐forming reactions and the TS structures have smaller cavity volumes than the reactants. Within the XP‐PCM formulation, molecules with larger volume experience a greater increase in the Pauli repulsion with the medium when the pressure increases. Therefore, the electronic energy of the reactants (with larger volume) increases faster than that of the TS (with smaller volume) with increasing pressure^[31]^ (see Equations. 24 and 25 in ref. [31]). This leads to reduced electronic energy barrier under pressure, i. e., negative $d{\Delta G}_{er}^{\neq }/dp$ , and therefore a negative contribution to ${\Delta V}^{\neq }$ . This phenomenon that molecules or atoms with more diffuse electron density experience greater repulsion under pressure has been noted previously.[^31^, ^76^, ^77^] To understand why $d{\Delta G}_{non-pV}^{\neq }/dp$ is also negative, we first note that, in general, the term $G_{non-pV}$ of the cavitation energy, which can be viewed as an entropic contribution, increases with the area of the cavity surface (see Equation (11) in ref. [31]) and the numerical density of the solvent (see Equation (12) in ref. [31]). Going from reactant to TS in the current reaction, the cavity size and surface area decrease, which leads to a negative ${\Delta G}_{non-pV}^{\neq }$ . With increasing pressure, ${\Delta G}_{non-pV}^{\neq }$ becomes more negative due to the increase of the numerical density of the solvent under higher pressure; therefore $d{\Delta G}_{non-pV}^{\neq }/dp$ is negative. This analysis shows that the change of cavity volume ${\Delta V}_{c}^{\neq }$ not only contributes directly to the activation volume, but also is used in the explanation of the effects of pressure on the changes of the electronic energy and entropy; thus, ${\Delta V}_{c}^{\neq }$ also contributes indirectly to activation volume.

## Conclusions

As part of our continuing efforts to apply a new quantum chemical method, the extreme pressure polarizable continuum model (XP‐PCM), to the study of reaction energy profiles under pressure and activation volumes,^[17]^ this work treats of the effects of pressure on the competing mechanisms of the thermal dimerization of 1,3‐cyclohexadiene and provides, for the first time, accurate, quantum chemical calculations of activation volumes. Gas‐phase ωB97XD and NEVPT2 calculations, based on the computed enthalpic barriers, suggest that the [4+2] cycloadduct ***endo*** **‐2** and the [6+4]‐ene adduct ***threo*** **‐4** are formed via concerted mechanisms, whereas the [4+2] cycloadduct ***exo*** **‐2** and the [2+2] cycloadducts ***syn*** **‐3** and ***anti*** **‐3** are formed via stepwise mechanisms. The reactions affording ***exo*** **‐2** and ***syn*** **‐3** share the same stepwise addition via **TS‐*meso***. The CCSD(T) activation enthalpies are in good agreement with experimental activation energies and previous calculations. A new [4+2]‐ene mechanism is considered in this work and found to be less favorable than the other mechanisms considered.

XP‐PCM calculations show that the reaction barriers of various dimerizations of 1,3‐cyclohexadiene decrease as the pressure increases, consistent with the expected negative activation volumes of these dimerizations. Pre‐TS minima, corresponding to van der Waals complexes, emerge under high pressures. The shift of the pre‐TS minimum toward the transition state is explained by the smaller volume and thus more favorable pV term of enthalpy of such complex compared with the separated reactants. The concerted [6+4]‐ene reaction shows a significant shift of the location of the transition state towards the reactant at high pressures due to a constant volume decrease during the transition state region. The comparison between the computed and experimental activation volumes shows good agreement and strongly indicates that the products ***endo*** **‐2** and ***threo*** **‐4** are compatible only with a concerted mechanism, while the product ***exo*** **‐2** is compatible only with a stepwise mechanism in common with the ***syn*** **‐3** product. The partition analysis of the activation volume shows that the change in the geometric, cavity volume of the molecule has the largest contribution but accounts for no more than 50 % of the whole activation volume; the change in the activation electronic energy and entropy at different pressures also play important roles in determining the value of activation volume.

This work demonstrates that accurate computation of activation volume is a powerful tool in deciphering competing reaction mechanisms in the current, and likely other reactions. The evolution of the cavity volume of the reactive system upon proceeding from the reactants to products, proves to be a useful diagnostic for analyzing the effect of the pressure on the reaction profiles. The analysis of partition of activation volume into physically meaningful contributions provides a new and extremely useful way to understand the origin of activation volume. The insights from such analysis will be useful in designing new high‐pressure reactions.

### Supporting Information

XP‐PCM parameters, 1 atm PES discussion, distortion‐interaction analysis, 3D drawings of transition state structures, T1 diagnostics, supplementary reaction profiles to Figure 6, activation volume calculations (PDF).

Raw output files of calculations containing optimized geometries and absolute energies (ZIP).

## Conflict of interest

The authors declare no conflict of interest.

1

## Supporting information

As a service to our authors and readers, this journal provides supporting information supplied by the authors. Such materials are peer reviewed and may be re‐organized for online delivery, but are not copy‐edited or typeset. Technical support issues arising from supporting information (other than missing files) should be addressed to the authors.

Supporting Information

## Data Availability

The data that support the findings of this study are openly available in ChemRxiv at https://doi.org/10.33774/chemrxiv‐2021‐dnhxj, reference number 0.
