# Supplementary material for: High‐Pressure Reaction Profiles and Activation Volumes of 1,3‐Cyclohexadiene Dimerizations Computed by the Extreme Pressure‐Polarizable Continuum Model (XP‐PCM)
Source: Chemistry. 2022 Apr 8;28(29):e202200246. doi: 10.1002/chem.202200246 (PMC9320931; doi:10.1002/chem.202200246)
Supplement: Supplementary file 1 — Supporting Information [file CHEM-28-0-s001.pdf]

Chemistry–A European Journal

Supporting Information

High-Pressure Reaction Profiles and Activation Volumes of 1,3-Cyclohexadiene Dimerizations Computed by the Extreme Pressure-Polarizable Continuum Model (XP-PCM)

Bo Chen,* K. N. Houk, and Roberto Cammi*

Table of Contents

The XP-PCM parameters 1
Potential energy surface at 1 atm 2
Distortion-interaction analysis 9
3D drawings of TS structures 11
T1 diagnostics of CCSD(T) calculations on closed-shell structures 12
Supplementary reaction profiles to Figure 6 13
Activation volume calculations at the ωB97XD/def2-TZVP level 14
Activation volume calculation at the B3LYP/6-31G(d) level 15
References 16
Coordinates of optimized structures 17

The XP-PCM parameters

The external medium transmitting the pressure is cyclohexane, which, at standard thermodynamic condition, has a relative dielectric constant ε_0=2.0165 and a mean valence electron density ρ=0.2004 eÅ^{-3}. The electronic energy G_{er}, and the pressure p have been computed with the solute embedded in a molecular cavity, enclosed in the solvent excluded surface (SES).[1] This SES cavity is formed by rolling a hard, probe sphere with the radius of the solvent molecule on the vdW surface of the solute. The vdW surface of the solute is the surface of a cavity formed by interlocking vdW spheres with scaled vdW radii centered on the nuclei of the solute. The standard vdW radii are multiplied by a scaling factor in the range f=1.2–0.95. This range of values of the scaling factor yield values of the pressure within the range p = 1–10 GPa. The Pauli repulsion barrier is located at the boundary of SES cavity and the barrier height Z_0 is computed using η=3 as value of Pauli semi-empirical parameter. The cavitation energy G_{cav} is computed using a vdW cavity with fixed scaling factor (f=1.2). The use of a fixed cavity for the calculation of G_{cav} is due to reasons of physical consistency with the Scaled Particle Theory method.[2]

Potential energy surface at 1 atm

Concerted [4+2] cycloadditions vs [6+4]-ene reactions. Figure S1 shows the potential energy surface of various dimerization pathways of 1,3-cyclohexadiene at 1 atm, calculated at the ωB97XD/def2-TZVP level of theory. Table S1 reports the computed reaction barriers at ωB97XD, multireference perturbation NEVPT2, and coupled-cluster CCSD(T) levels.

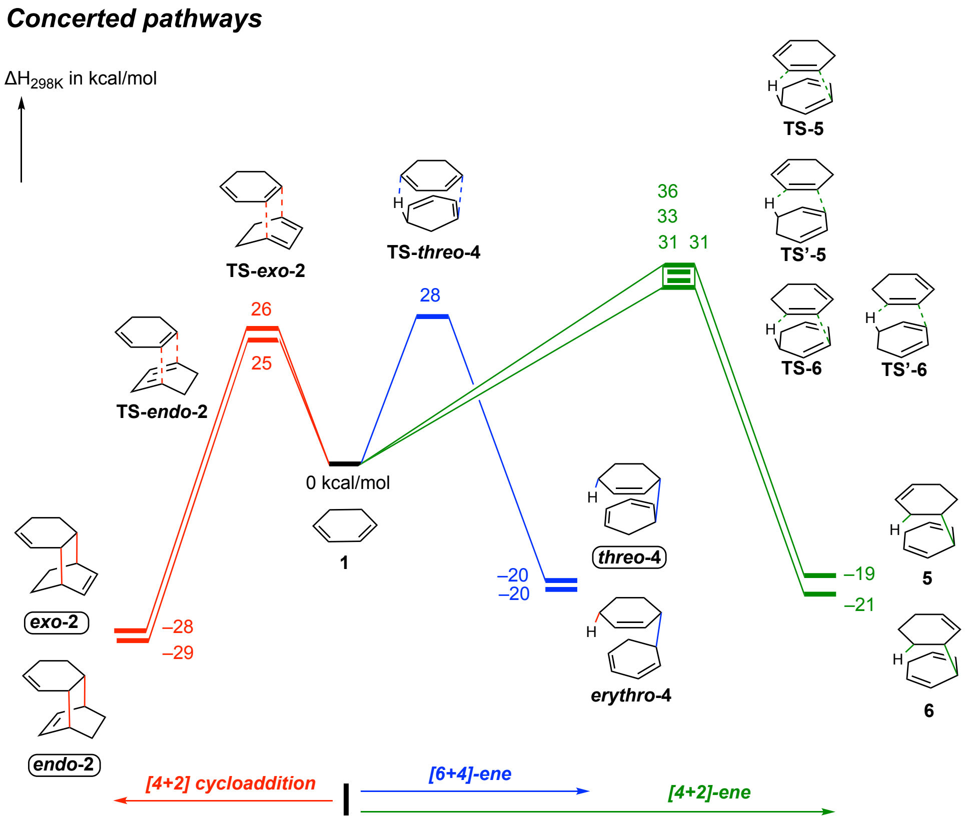

Figure S1. 1 atm enthalpic profiles of concerted pathways calculated at the ωB97XD/def2-TZVP level of theory. Experimentally-observed dimers are indicated by rounded-corner boxes around the labels.

At the ωB97XD/def2-TZVP level of theory, the *endo* [4+2] cycloaddition pathway going through **TS-*endo*-2** is calculated to have the lowest enthalpic barrier of 25 kcal/mol. The barrier of the *exo* pathway is 1 kcal/mol higher, likely due to the absence of secondary orbital interactions[3] in **TS-*exo*-2**. ωB97XD/def2-TZVP calculations also show that the activation enthalpy of the *threo* [6+4]-ene reaction going through **TS-*threo*-4** is 3 kcal/mol higher than that of the *endo* [4+2] cycloaddition. However, CCSD(T)/def2-TZVP single-point calculations

give opposite results that the *threo* [6+4]-ene pathway is the most favored pathway, with **TS-*threo*-4** being 1 kcal/mol lower than **TS-*endo*-2**. The CCSD(T) computed activation enthalpies agree excellently with the experimental activation energies (Table S1).

Table S1. Calculated activation enthalpies $\Delta H^{\ddagger}_{298K}$ and Gibbs activation energy $\Delta G^{\ddagger}_{298K}$ (in parenthesis), and experimental activation energies E_a and pre-exponential factor lgA of the Arrhenius equation for the five dimerization mechanisms of cyclohexadiene. Zero-point vibrational, thermal, and entropic corrections are computed at the ωB97XD/def2-TZVP level. For the stepwise TS structures, the arrow "→" points from spin-unprojected to spin-projected energies. All energies are in kcal/mol.

Mechanism	TS structure	Calc. $\Delta H^{\ddagger}_{298K}$ ($\Delta G^{\ddagger}_{298K}$) in kcal/mol			Expt. E_a in kcal/mol	Expt. lgA
		ωB97XD[a]	NEVPT2[b]	CCSD(T)[c]		
Concerted [4+2]	**TS-*endo*-2**	25 (38)	18 (31)	22 (34)	22.6	5.46
Concerted [4+2]	**TS-*exo*-2**	26 (40)	20 (34)	24 (37)	n.a.	n.a.
Concerted [6+4]-ene	**TS-*threo*-4**	28 (42)	18 (33)	21 (35)	21.5	3.87
Stepwise	**TS-meso**	24 (36) → 18 (30)[d]	19 (31)		24.2/24.3	5.51/5.10
Stepwise	**TS-*rac***	28 (40) → 21 (34)[e]	24 (37)		25.3	5.52

[a] (U)ωB97XD/def2-TZVP; [b] NEVPT2(8,8)/def2-TZVP//(U)ωB97XD/def2-TZVP; [c] CCSD(T)/def2-TZVP//ωB97XD/def2-TZVP; [d] $\langle S^2\rangle=0.62$; [e] $\langle S^2\rangle=0.73$;

Interestingly, although both experiments and calculations show that the concerted [6+4]-ene reaction (via **TS-*threo*-4**) has the lowest activation energy/enthalpy, the corresponding [6+4]-ene adduct ***threo*-4** is only a minor product, comprising 8% of the observed dimers. Importantly, the product ratio does not change over the reaction course and all dimers are verified to be kinetically persistent, i.e., no interconversion among the dimers.[4] Therefore, the low product ratio of ***threo*-4** must mean that the [6+4]-ene reaction is relatively slow. Since the rate of a reaction is determined by $\Delta G^{\ddagger}$, not just by $\Delta H^{\ddagger}$ or E_a, the two observations—the lowest activation energy/enthalpy and a relatively slow reaction rate—about the [6+4]-ene reaction can be reconciled by realizing that this reaction has a significantly smaller activation entropy, compared with other reactions. Calculations show that the entropy of **TS-*threo*-4** is 5 J/mol·K smaller than **TS-*endo*-2**; this entropic difference corresponds to a difference of 1.5 kcal/mol in $\Delta G^{\ddagger}_{298K}$, in favor of **TS-*endo*-2**. Inclusion of the entropic contribution to the Gibbs activation energy reverses the kinetic preference of [6+4]-ene

reaction and [4+2] cycloaddition. The smaller entropy of **TS-*threo*-4** indicates higher structural order, a feature that also correlates with the large negative activation volume of the [6+4]-ene reaction, discussed in the main text. The difference in activation entropy is also reflected in the pre-exponential factor of the Arrhenius equations for these reactions, according to the thermodynamic formulation of the transition state theory.[5–8] Consistent with the computed smaller entropy of **TS-*threo*-4**, the [6+4]-ene reaction was experimentally measured to have a significantly smaller pre-exponential factor of the Arrhenius equation (lgA = 3.87), compared with other reactions (lgA > 5).

The [4+2] cycloadducts are computed to be the most thermodynamically stable ones among the 1,3-cyclohexadiene dimers considered. The [6+4]-ene adduct ***threo*-4** is about 10 kcal/mol less stable, but was observed to be kinetically persistent at 70 °C up to 3 kbar as well as at 110 °C and 1 atm.[4] At elevated temperature (161 °C), ***threo*-4** does slowly disproportionate into benzene and cyclohexene; a retro-[4+2]-ene mechanism was proposed for this disproportionation.[4] Our calculations (Figure S2) show that this disproportionation has a high enthalpic barrier of 39 kcal/mol, consistent with the observed, very slow disproportionation of ***threo*-4**, only at elevated temperatures.

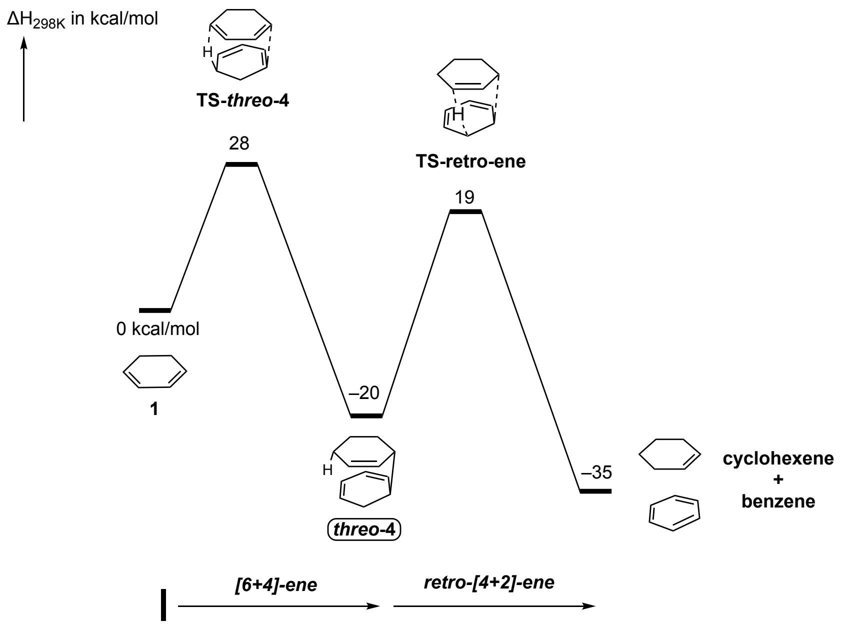

Figure S2. 1 atm enthalpic profiles of the retro-[4+2]-ene reaction following the [6+4]-ene reaction, calculated at the ωB97XD/def2-TZVP level of theory. This retro-[4+2]-ene is calculated to have an enthalpic barrier of 39 kcal/mol, with respect to ***threo*-4**, at both the ωB97XD/def2-TZVP and CCSD(T)/def2-TZVP//ωB97XD/def2-TZVP levels of theory.

The TS structure of a second concerted [6+4]-ene pathway, **TS-*erytho*-4**, was not located on the PES. Attempts by scanning the C1–C1’ and/or C5–H/C4’–H bonds (see Figure S3 for the labeling) and using the highest energy structure in the scan as the initial structure for TS optimization lead to either the [4+2] cycloaddition TS structure **TS-*exo*-2** or the [4+2]-ene TS structure **TS-6**, both with lower energies than the initial structure. Figure S3 illustrates the geometric similarity of the three structures; **TS-*erytho*-4** and **TS-*exo*-2** share the same forming C1–C1’ bond while **TS-*erytho*-4** and **TS-6** share the same C5–H···C4’ H-transfer moiety. The existence of the two lower energy TS structures near **TS-*erytho*-4** on the PES likely makes **TS-*erytho*-4** no longer a stationary point. These calculations are consistent with the [6+4]-ene adduct ***erytho*-4** not being observed experimentally.

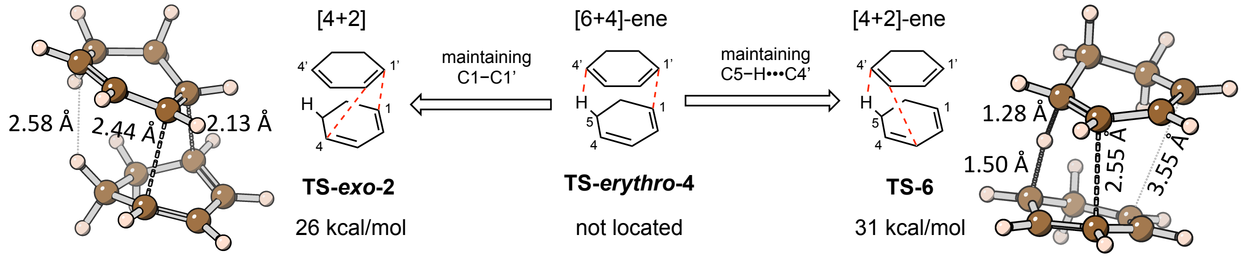

Figure S3. The structural relationship among three similar TS structures. ωB97XD/def2-TZVP enthalpies (ΔH_{298K}) of the optimized structures are shown.

Concerted [4+2]-ene pathways. As shown in Figure S1, the computed enthalpic barriers of [4+2]-ene reactions are 2–11 kcal/mol higher than those of the [4+2] cycloaddition and [6+4]-ene pathways. A plausible reason for the higher energies of the [4+2]-ene TS structures is that the conjugation between the dienes in the bottom ring (Figure S1) is lost in the [4+2]-ene TS structures whereas it remains in the TS structures of the [4+2]-cycloadditions and [6+4]-ene reaction. These >30 kcal/mol large barriers of the [4+2]-ene reactions are consistent with the fact that [4+2]-ene products are not observed experimentally.

Stepwise pathways. Figure S4 shows the 1 atm enthalpic reaction profiles of the stepwise pathways. The initial C–C bond formation between two molecules of **1**, via the *rac* addition (the left branch) or the *meso* addition (the right branch), leads to a diradical intermediate ***rac*-8** or ***meso*-8**. **TS-*meso*** is computed to be 4 kcal/mol lower in enthalpy than **TS-*rac***; ***rac***-8 and ***meso*-8** are computed to be similar in enthalpy. ***rac*-8** and ***meso*-8** have *anti* conformations around the forming C–C bonds. The search for stepwise TS structures in *gauche*

conformations lead to concerted TS structures. For example, the two *gauche* conformations of **TS-*rac*** evolved into **TS-*endo*-2** and **TS-*threo*-4** during TS optimization. It seems that only the *anti* conformations of the stepwise TS structures are stationary points on the PES at the ωB97XD/def2-TZVP level of theory. In the second step, two TS structures starting from ***rac*-8** (or ***meso*-8**) were located, affording [2+2]-cycloadduct ***anti*-3** (or ***syn*-3**) and [4+2]-cycloadduct ***endo*-2** (or ***exo*-2**). The [4+2] cycloadducts are computed to be about 8-11 kcal/mol more stable than the [2+2] cycloadducts.

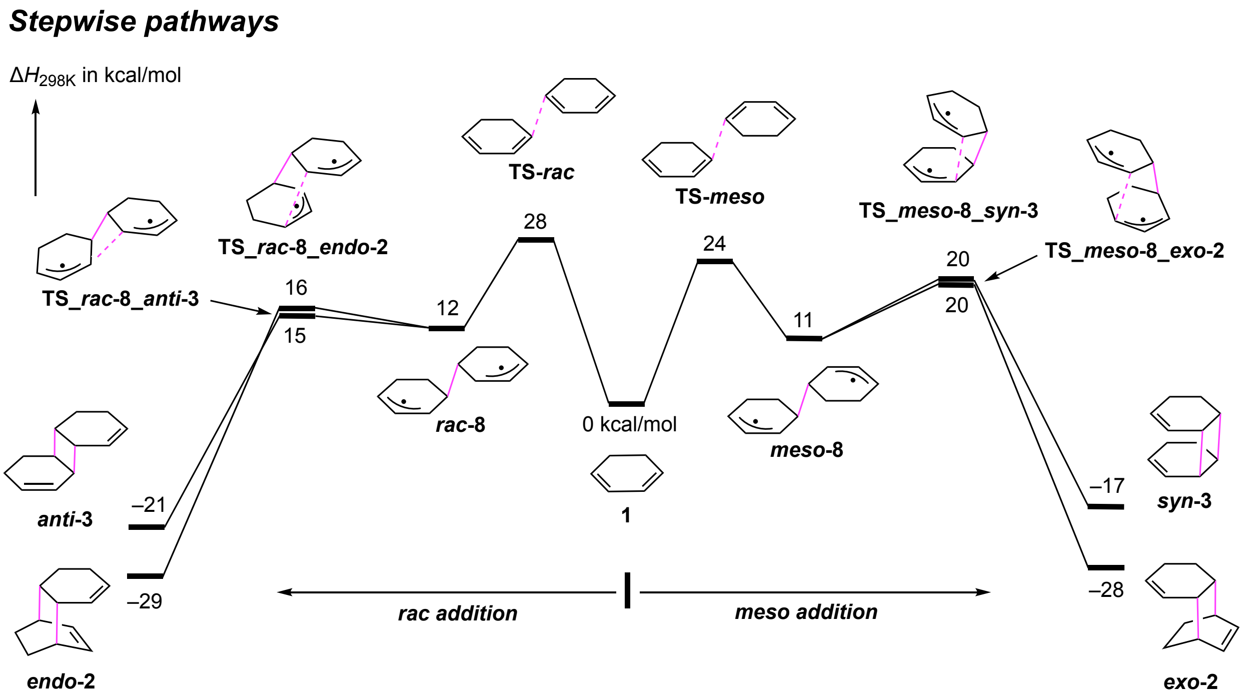

Figure S4. 1 atm enthalpic reaction profiles of stepwise pathways of 1,3-cyclohexadiene dimerization, calculated at the ωB97XD/def2-TZVP level of theory.

Formations of ***exo*-2** and ***syn*-3** share the same stepwise *meso* addition mechanism. Their respective TSs (**TS_*meso*-8_*exo*-2** and **TS_*meso*-8_*syn*-3**) for the second bond formation from the same intermediate ***meso*-8** were computed to have the same enthalpy of 20 kcal/mol. From the *anti* conformation of ***meso*-8**, more rotation about the first formed C—C bond (~180 degrees, see Figure 3 in the main text) is necessary in order to reach a geometry for the second bond to form to give ***syn*-3**. This might be the reason that ***syn*-3** has a lower product ratio than ***exo*-2** in experiment.

Many structures in the stepwise pathways are open-shell singlet states with significant multi-reference character. Single-reference DFT calculations using the broken-symmetry approach, which was what we used to compute the energies of, for example, **TS_*meso*** and **TS_*rac*** (Table S1), introduce spin contamination from the triplet to the open-shell singlet wave functions, as indicated by the $<S^2>$ values from broken-symmetry DFT calculations, for instance $<S^2> = 0.62$ for **TS-*meso***. The spin-contamination can be removed by the Yamaguchi and Houk spin-projection procedure.[9]

However, either the spin-contaminated or the spin-projected energies shows that the stepwise **TS-*meso*** is lower in energy than the concerted **TS-*endo*-2**, in contradiction to the experimental activation energies E_a in Table S1. In order to compare the computed barriers of the stepwise pathways with the concerted pathways at a consistent level of theory, we resorted to NEVPT2 calculations. These NEVPT2 calculations are based on CASSCF(8,8) wavefunctions and, in addition to the fully-accounted dynamic electron correlation within the active space, the dynamic electron correlation outside of the active space is recovered through the 2nd order perturbation theory. As shown in Table S1, **TS-*meso*** was computed to be 1 kcal/mol higher in enthalpy than **TS-*endo*-2** at the NEVPT2(8,8)/def2-TZVP//(U)ωB97XD/def2-TZVP level, consistent with the experimental E_a. However, the 3-5 kcal/mol difference between **TS-*meso*** and **TS-*rac*** at ωB97XD and NEVPT2 levels is larger than the experimental E_a, though this large difference is consistent with previous calculations.[10]

The [4+2] cycloadducts ***endo*-2** and ***exo*-2** may be formed by either a concerted or stepwise mechanism as shown in Figure 3 in the main text. The NEVPT2 calculations in Table S1 suggest that these two [4+2]-cycloadducts are formed by different mechanisms. For ***endo*-2**, the concerted mechanism is computed to be enthalpically more favorable whereas for ***exo*-2**, the stepwise mechanism is more favorable.

Although NEVPT2 calculations distinguishes the concerted and stepwise pathways reasonably well, they underestimate the enthalpic barriers of most pathways by about 4 kcal/mol, when compared against the experimental activation energy E_a (Table S1). We recommend referring to CCSD(T) barriers for the comparison with experimental E_a, and to the NEVPT2 barriers for comparing relative barrier heights, especially between the concerted and stepwise pathways. Irrespective of the level of theory, the concerted and stepwise pathways were computed to have enthalpic barriers within 5 kcal/mol. The small enthalpic difference

between these TS structures are consistent with the experiment, confirming the competitive nature of the concerted and stepwise mechanisms in this reaction.

We summarize our calculations and discussions on the 1 atm PES of the thermal dimerization of cyclohexadiene as follows:

- For ***endo*-2**, the concerted [4+2] cycloaddition is computed to be more favorable than the stepwise mechanism at both the ωB97XD (spin-projected) and NEVPT2(8,8) levels. The CCSD(T) computed $\Delta H^{\ddagger}_{298K}$ for the concerted [4+2] cycloaddition gives the best agreement with the experimental E_a.

- For ***exo*-2**, the stepwise mechanism through the ***meso*-8** intermediate is supported against the concerted [4+2] cycloaddition by both the ωB97XD and NEVPT2(8,8) calculations.

- For ***threo*-4**, the concerted [6+4]-ene mechanism has the lowest $\Delta H^{\ddagger}_{298K}$ among all the reactions considered, as shown by the NEVPT2 and CCSD(T) calculations. The CCSD(T) $\Delta H^{\ddagger}_{298K}$ for this reaction is in excellent agreement with the experimental E_a.

- For ***syn*-3**, the thermally allowed stepwise mechanism through the ***meso*-8** intermediate is favored over the concerted mechanism by both the ωB97XD (spin-projected) and NEVPT2(8,8) calculations; however, both methods give an underestimation (5-6 kcal/mol) of the experimental activation energy.

- For ***anti*-3**, similar as ***syn*-3**, only the stepwise mechanism through the ***rac*-8** intermediate is thermally allowed. Only the NEVPT2(8,8) calculations give a reasonable estimation of the experimental activation energy.

- The above calculations are in good agreement with the previous calculations by Ess *et al.*[10] In addition, we explored the plausible [4+2]-ene mechanisms that were not considered before. The large computed barriers (> 31 kcal/mol at ωB97XD/def2-TZVP level of theory) are consistent with the fact that the corresponding [4+2]-ene products **5** and **6** were not observed experimentally.

Distortion-interaction analysis

The distortion-interaction analysis of the transition states is given in Figure S5. Two factors are in play here: for the concerted [4+2] TSs, **TS-*endo*-2** is less distorted with slightly larger interaction energy than **TS-*exo*-2**. As can be seen in Figure S5, the ethano hydrogens are near the diene in the exo TS, and a distortion of the bottom diene is required to avoid van der Waals repulsion. At the same time, the overlap of diene and alkene orbitals is diminished. In addition, 2nd-order orbital interaction is available in the *endo* but not the *exo* TS structures.

Concerted [4+2]-cycloaddition **Concerted [6+4]-ene**

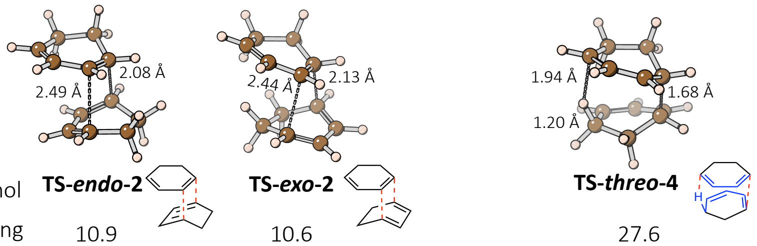

Energies in kcal/mol	TS-*endo*-2	TS-*exo*-2	TS-*threo*-4
Distortion energy of the top ring	10.9	10.6	27.6
Distortion energy of the bottom ring	19.6	20.6	27.4
Total distortion energy	30.5	31.2	55.0
Interaction energy	−9.8	−8.3	−33.6
$\Delta E^{\ddagger}$	20.7	22.9	21.4

Concerted [4+2]-ene

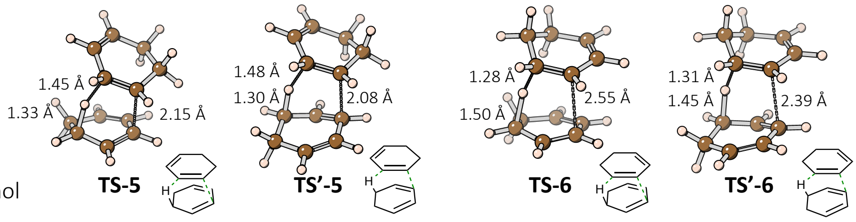

Energies in kcal/mol	TS-5	TS'-5	TS-6	TS'-6
Distortion energy of the top ring	14.9	13.0	11.5	10.7
Distortion energy of the bottom ring	32.0	30.1	42.4	38.0
Total distortion energy	46.9	43.1	53.9	48.7
Interaction energy	−10.3	−9.8	−21.5	−17.5
$\Delta E^{\ddagger}$	36.6	33.3	32.4	31.2

Figure S5. Distortion-interaction analysis on the concerted TSs at the CCSD(T)/def2-TZVP//ωB97XD/def2-TZVP level of theory.

The distortion energies of the top rings are very similar for all these TSs except for **TS-*threo*-4**, whose distortion energy of the top ring (27.6 kcal/mol) is >10 kcal/mol higher. The similar distortion energy of the top rings in the [4+2]-cycloaddition and [4+2]-ene TSs can be explained by noticing that the top ring always participates the reaction as an alkene, the 2p-component in either the [4+2]-cycloaddition or [4+2]-ene reaction. In contrast, the top ring in

the [6+4]-ene **TS-*threo*-4** participates the reaction as a diene, the 4p -component, so its distortion energy is expected to be different.

Even more importantly, for all the ene TSs, the distortion energies of the bottom rings are much larger than those of the bottom rings of the [4+2]-cycloaddition TSs. This large distortion energy arises from the H transfer nature of the ene TS. There is a correlation with the distance of the C–H bond that is breaking in the ene reaction—the longer the C–H distance, the larger the distortion energy of the bottom ring. For example, this C–H distance is 1.50 Å in **TS-6**, the longest among the ene TSs; correspondingly, the distortion energy of the bottom ring in **TS-6** is 42.4 kcal/mol, also the largest among the ene TS. In contrast, the shortest C–H distance (1.20 Å) and the smallest distortion energy (27.4 kcal/mol) of the bottom ring come from the [6+4]-ene TS, **TS-*threo*-4**.

For the interaction energies, **TS-*threo*-4** has the largest one (–33.6 kcal/mol), likely due to the very short distance of the forming C–C bond (1.68 Å). This short C–C bond necessitates a larger extent of pyramidalization of the corresponding sp^2 carbons in the reactants, which would lead to higher distortion energies. The interaction energies in the [4+2]-ene TSs seem to correlate with the distance of the forming C–H bonds—the short this C–H distance, the larger the interaction energy.

Overall, the [4+2]-cycloaddition TS **TS-*endo*-2** has the smallest total distortion energy and a reasonable interaction energy, leading to the smallest electronic energy barrier of this pathway.

3D drawings of TS structures

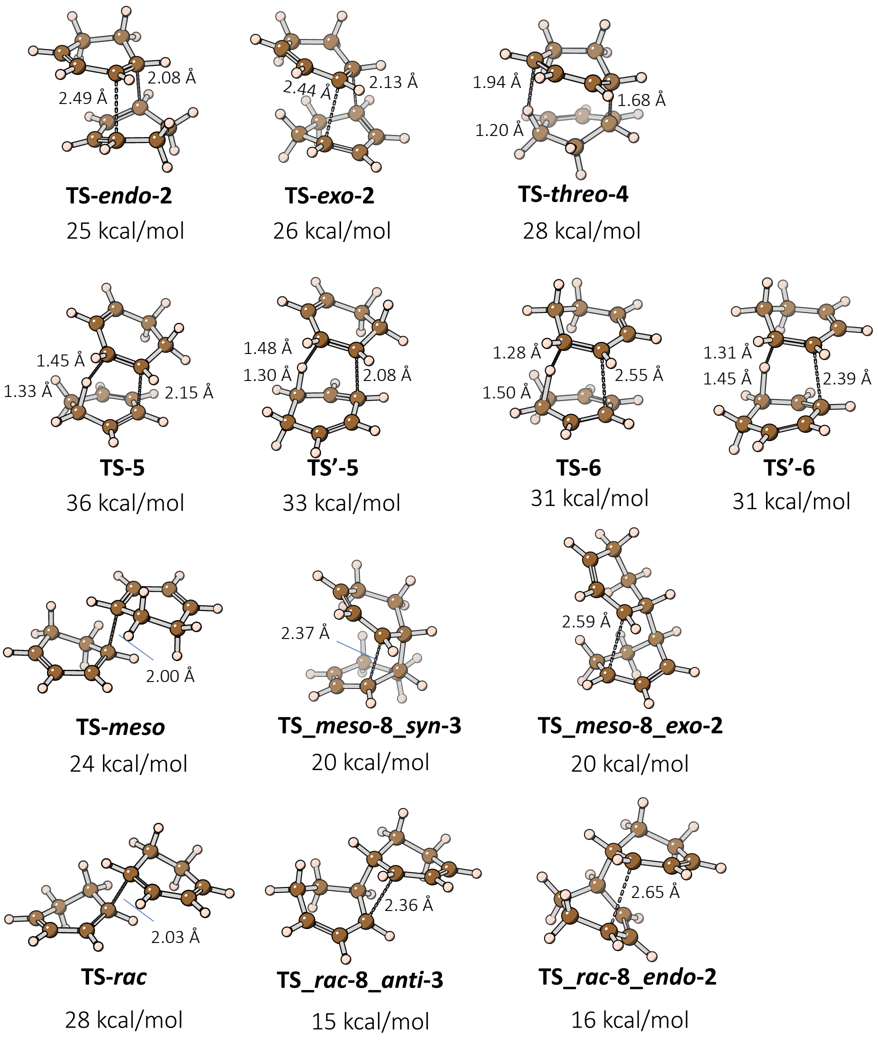

Figure S6. TS structures and $\Delta H^{\ddagger}_{298K}$ computed at the ωB97XD/def2-TZVP level of theory.

T1 diagnostics of CCSD(T) calculations on closed-shell structures

**Structure**	T1 diagnostic
**1**	0.01080592
**5**	0.01060243
**6**	0.01058748
**TS’-5**	0.01094374
**TS’-6**	0.01077051
**TS-5**	0.01085802
**TS-6**	0.01104191
**TS-*endo*-2**	0.01054478
**TS-*exo*-2**	0.01068457
**TS-retro-ene**	0.01063140
**TS-*threo*-4**	0.01104525
**benzene**	0.01022066
**cyclohexene**	0.00985970
***endo*-2**	0.01041448
***erythro*-4**	0.01066312
***exo*-2**	0.01047967
***threo*-4**	0.01068405

Supplementary reaction profiles to Figure 6

Comparison of the profiles in Figure S7 with the corresponding ones in Figure 6 in the main text show that the profiles of *endo* and *exo* [4+2]-cycloadditions have similar behavior under pressure, so do the *meso* and *rac* stepwise additions.

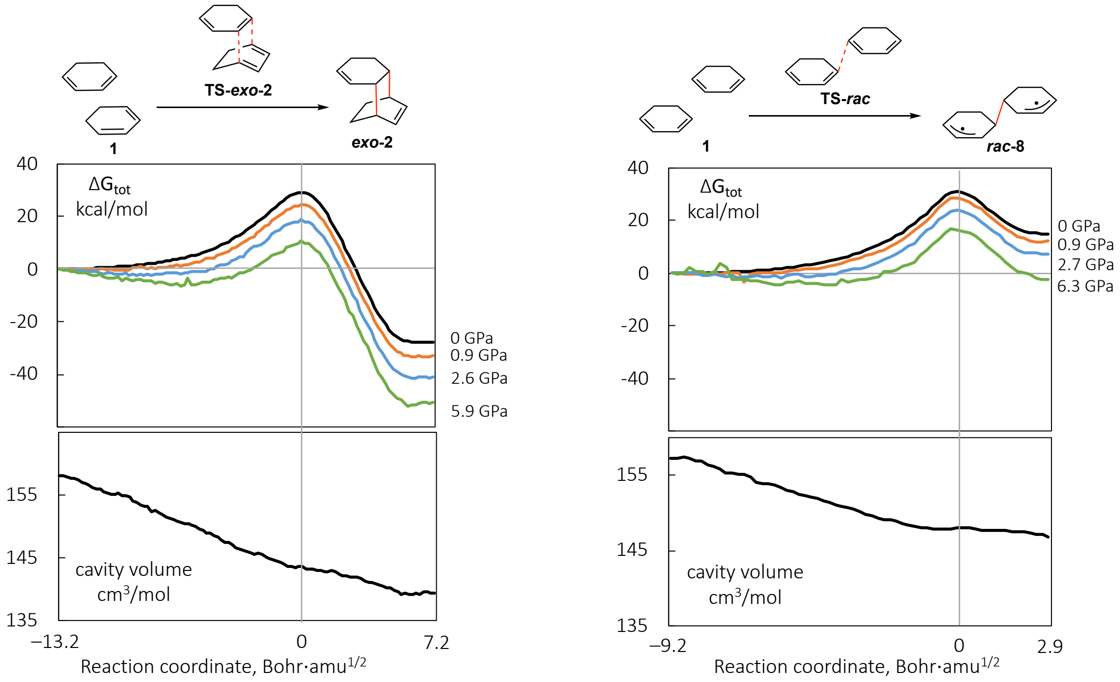

Figure S7. Effective reaction profiles at different pressures and cavity volume profiles (vdW cavity with a scaling factor of 1.2 of the Bondi radii) of concerted *exo*-[4+2] cycloaddition, and the first step of the *rac* stepwise addition of cyclohexadiene dimerization, calculated by the XP-PCM method at the ωB97XD/def2-TZVP level.

Activation volume calculations at the ωB97XD/def2-TZVP level

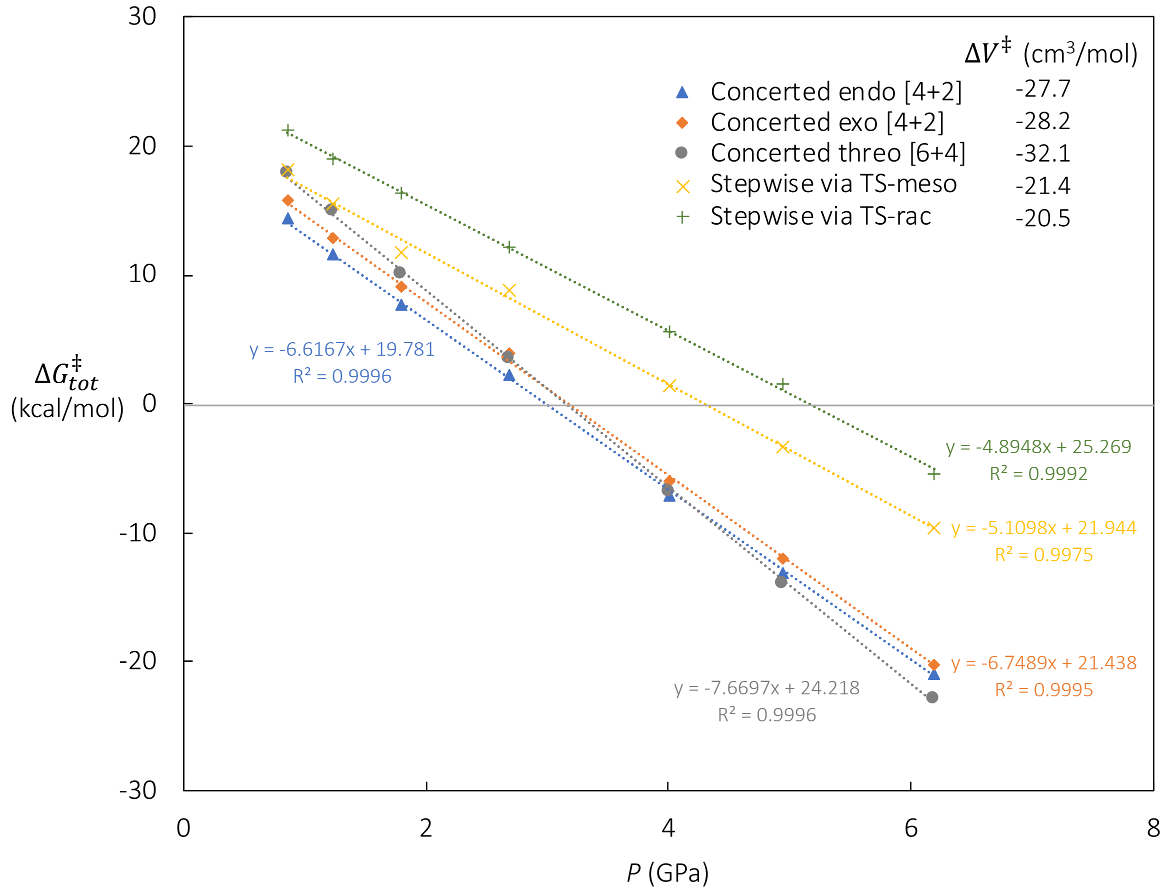

Figure S8. Plots of computed (ωB97XD/def2-TZVP) reaction barriers at different pressures $\Delta G^{\ddagger}_{tot}(p)$ for the five reactions of 1,3-cyclohexadiene. The slope of a curve is the activation volume $\Delta V^{\ddagger}$ of the corresponding reaction. 1 kcal/mol/GPa = 4.184 cm^3/mol. Activation volumes computed at B3LYP/6-31G(d) level (Figure S9) are about 0.2–1.5 cm^3/mol smaller, but the trend remains the same.

Activation volume calculation at the B3LYP/6-31G(d) level

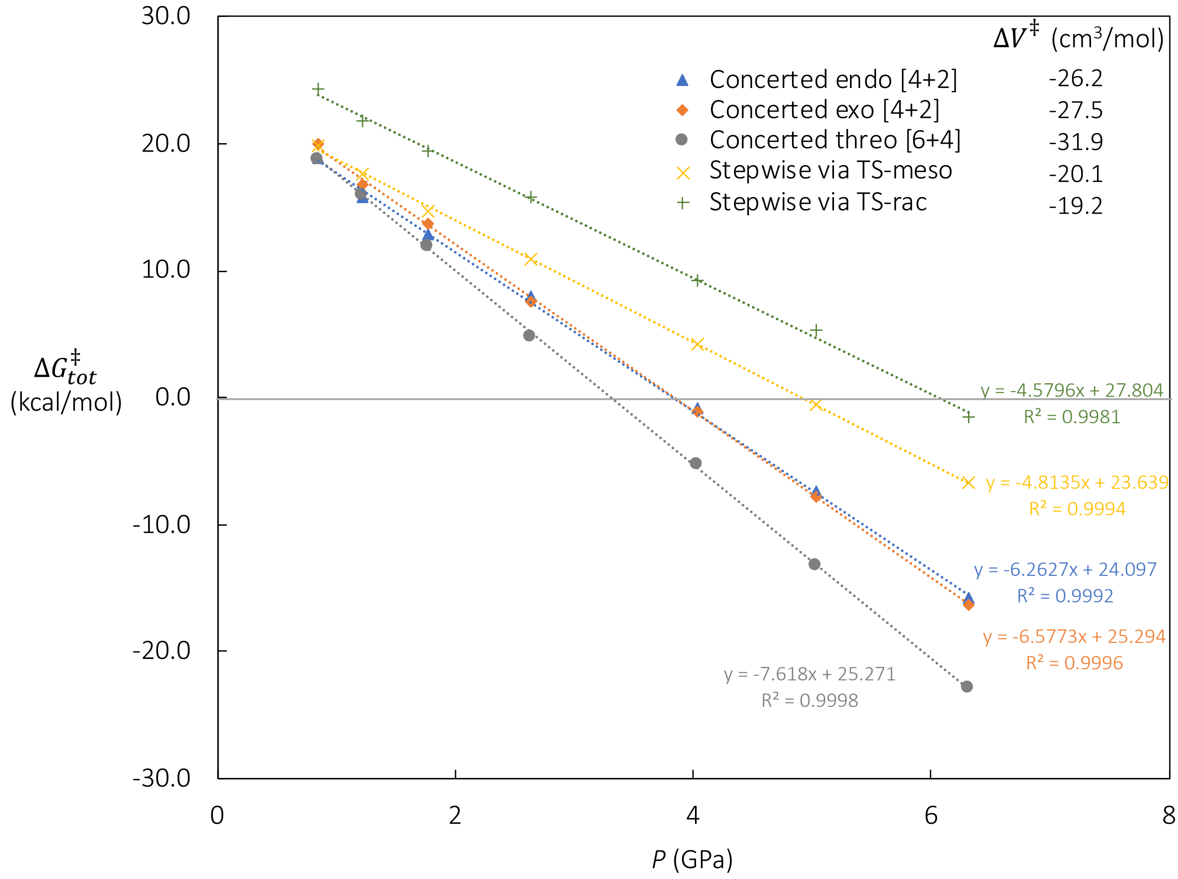

Figure S9. Plots of computed (B3LYP/6-31G*) reaction barriers at different pressures $\Delta G^{\ddagger}_{tot}(p)$ for the five reactions of 1,3-cyclohexadiene. The slope of a curve is the activation volume $\Delta V^{\ddagger}$ of the corresponding reaction. 1 kcal/mol/GPa = 4.184 cm^3/mol.

References

[1] F. M. Richards, *Annu Rev Biophys Bio* **1977**, *6*, 151–176.

[2] R. A. Pierotti, *Chem Rev* **1976**, *76*, 717–726.

[3] R. Hoffmann, R. B. Woodward, *J Am Chem Soc* **1965**, *87*, 4388–4389.

[4] F. Klärner, B. M. J. Dogan, O. Ermer, W. von E. Doering, M. P. Cohen, *Angewandte Chemie Int Ed Engl* **1986**, *25*, 108–110.

[5] S. Glasstone, K. J. Laidler, H. Eyring, *The Theory of Rate Processes: The Kinetics of Chemical Reactions, Viscosity, Diffusion and Electrochemical Phenomena*, McGraw-Hill Book Company, New York, **1941**.

[6] J. I. Steinfeld, J. S. Francisco, W. L. Hase, *Chemical Kinetics and Dynamics*, Upper Saddle River, N.J. : Prentice Hall, **1999**.

[7] H. Eyring, S. H. Lin, S. M. Lin, *Basic Chemical Kinetics*, Wiley, New York, **1980**.

[8] K. J. Laidler, *Chemical Kinetics, 3rd Edition*, Pearson, **1987**.

[9] K. Yamaguchi, Y. Takahara, T. Fueno, K. N. Houk, *Theor Chim Acta* **1988**, *73*, 337–364.

[10] D. H. Ess, A. E. Hayden, F.-G. Klärner, K. N. Houk, *J Org Chem* **2008**, *73*, 7586–7592.

Coordinates of optimized structures

The coordinates and energies of optimized structures are also available as Gaussian output files.

1 (1,3-cyclohexadiene)
scf done: -233.423357

C	-0.109985	0.723428	-1.249442
C	-0.063317	1.414874	-0.111565
C	0.248862	0.722470	1.186368
C	-0.248862	-0.722470	1.186368
C	0.063317	-1.414874	-0.111565
C	0.109985	-0.723428	-1.249442
H	1.337820	0.736855	1.330758
H	-0.290658	1.223849	-2.193127
H	0.179600	-1.267072	2.029129
H	0.190118	-2.491043	-0.112309
H	0.290658	-1.223849	-2.193127
H	-0.190118	2.491043	-0.112309
H	-0.179600	1.267072	2.029129
H	-1.337820	-0.736855	1.330758

5
scf done: -466.880966

C	0.072029	2.212818	1.655073
H	0.574441	3.117998	2.013530
C	0.547040	-0.283561	3.240675
H	-0.115067	-1.127045	3.014582
C	-0.943737	1.738492	2.700624
C	1.332382	0.081932	2.017519
H	-1.335014	2.625273	3.207853
H	2.103648	-0.610056	1.695644
C	-2.252811	0.164103	-0.270904
H	-1.983524	-0.615922	-0.975063
C	-1.889320	0.045996	0.997763
H	-1.332384	-0.831974	1.298657
H	1.224834	-0.651498	4.015958
C	-0.250659	0.900982	3.780374
H	0.439865	1.560905	4.314300
H	-0.985467	0.559952	4.514129
C	-3.037971	1.319227	-0.807820
H	-2.446877	1.859422	-1.558270
H	-3.913303	0.949208	-1.354546
C	-3.476965	2.260221	0.269672
H	-0.451314	2.510866	0.741729
H	-4.130123	3.076062	-0.021427
C	-3.118007	2.129987	1.537824
H	-3.481064	2.846278	2.268907
C	-2.195061	1.063746	2.061132
H	-2.708106	0.550327	2.885631
C	1.116922	1.188585	1.319300
H	1.728968	1.390506	0.446135

6
scf done: -466.885127

C	0.969222	0.938197	0.437493
C	2.587005	-0.990607	-0.870740
H	0.512257	1.531924	1.231396
H	3.141087	-1.685941	-1.493119
C	-2.710803	0.665937	1.132661
H	-3.272442	1.151964	1.923433
C	-1.707546	-0.140762	1.443663
H	-1.455068	-0.301686	2.487779
C	-1.307648	-0.589103	-0.969615
H	-0.751586	-1.085886	-1.757948
C	-0.857402	-0.859539	0.437221

anti-3
scf done: -466.885678

C	-0.105591	0.765228	-0.509832
C	1.472443	1.202338	1.438746
C	0.105591	0.780291	1.024317
C	0.823877	1.642446	-1.325418
C	2.284053	1.503071	-0.901325
C	2.446659	1.511152	0.588787
H	-3.421938	-1.773800	0.984859
H	-2.707801	-0.573419	-1.301067
H	1.678626	1.225988	2.504755
H	-0.655364	1.314761	1.596120
H	-2.881760	-2.302424	-1.345707

H	-0.957652	-1.938657	0.621019
C	1.510008	-1.415009	-0.223207
H	1.211030	-2.455990	-0.314568
C	0.646466	-0.540848	0.646173
H	0.864906	-0.797135	1.692044
C	3.100681	0.417376	-0.784712
H	2.888442	0.936495	-1.726846
H	4.189902	0.404525	-0.690625
C	2.473898	1.173989	0.383040
H	2.927315	0.830145	1.318306
H	2.689794	2.241100	0.300505
C	-3.133149	0.951907	-0.274802
H	-3.076886	2.030596	-0.466804
H	-4.192982	0.699870	-0.401824
C	-2.312223	0.214128	-1.286433
H	-2.573048	0.358246	-2.329661
H	0.516642	1.270657	-0.502362

H	0.519251	2.685488	-1.196511
H	0.718153	1.416748	-2.390239
H	2.881760	2.302424	-1.345707
H	2.707801	0.573419	-1.301067
H	-0.718153	-1.416748	-2.390239
H	3.421938	1.773800	0.984859
C	0.105591	-0.765228	-0.509832
C	-1.472443	-1.202338	1.438746
C	-0.105591	-0.780291	1.024317
C	-0.823877	-1.642446	-1.325418
C	-2.284053	-1.503071	-0.901325
C	-2.446659	-1.511152	0.588787
H	1.144122	-1.006261	-0.743863
H	-0.519251	-2.685488	-1.196511
H	-1.678626	-1.225988	2.504755
H	0.655364	-1.314761	1.596120
H	-1.144122	1.006261	-0.743863

benzene
scf done: -232.246598

C	-2.363627	3.339861	-0.001276
C	-3.396218	2.471671	0.322055
C	-3.335735	1.143673	-0.074638
C	-2.242710	0.683834	-0.794646
C	-1.210118	1.552024	-1.117977
C	-1.270601	2.880022	-0.721284
H	-4.141732	0.465993	0.177736
H	-2.410792	4.376444	0.308340
H	-2.195545	-0.352749	-1.104262
H	-0.356929	1.193139	-1.679985
H	-0.464605	3.557703	-0.973657
H	-4.249408	2.830555	0.884063

cyclohexene
scf done: -234.657602

C	-2.551257	-0.753832	0.335673
C	-1.085566	-0.679041	0.014711
C	-0.579887	0.761412	-0.015094
C	-1.117511	1.548564	1.175808
C	-2.643165	1.602838	1.145200
C	-3.246975	0.265404	0.823418
H	0.511724	0.777577	-0.027088
H	-0.524333	-1.258868	0.757091
H	-0.896743	-1.166164	-0.945813
H	-3.051221	-1.698931	0.149890
H	-0.793435	1.060924	2.100828
H	-0.704500	2.559150	1.188398
H	-3.028444	1.955900	2.105646
H	-2.978080	2.337104	0.402909
H	-4.309400	0.144331	1.008583
H	-0.915321	1.241301	-0.940118

endo-2
scf done: -466.899749

C	0.291810	0.530368	1.380243
C	2.562708	-0.975404	0.295161
H	0.112664	1.457876	1.931430
H	3.424087	-1.521847	-0.073744
C	-0.149292	0.735376	-1.094878
H	0.260104	1.047992	-2.056143
C	-0.569233	-0.708094	-1.102564
H	-0.463934	-1.332203	-1.980200
C	-1.111639	-0.080418	1.126425
H	-1.520494	-0.465147	2.061715
C	-1.047590	-1.137156	0.059154
H	-1.387516	-2.150225	0.234091
C	2.149661	0.107425	-0.347541

erythro-4
scf done: -466.883372

C	2.226775	1.512704	-1.564475
C	0.716845	-0.693655	-0.207095
H	3.218779	1.739403	-1.964365
H	1.216505	-1.664080	-0.332768
C	-0.883289	0.734630	2.507341
H	-1.061361	1.770305	2.772235
C	-0.381598	-0.114877	3.399362
H	-0.138642	0.213912	4.402317
C	-0.225805	-1.915420	1.770674
H	-0.005172	-2.945672	1.512106
C	-0.138614	-1.510053	3.036128
H	0.137538	-2.210161	3.815593
C	1.681126	0.220808	0.498368

H	2.681238	0.435880	-1.236343	H	1.832816	0.041566	1.556946
C	0.930190	0.906840	0.013993	C	2.332576	1.211845	-0.096952
H	1.209385	1.965641	0.027057	H	2.991930	1.840840	0.492439
C	1.881866	-1.477843	1.531688	C	0.366656	-0.168692	-1.600788
H	1.154187	-2.250728	1.263095	H	-0.360377	0.645672	-1.516834
H	2.608020	-1.958047	2.191896	H	-0.117194	-0.961685	-2.176652
C	1.199251	-0.327892	2.264477	C	1.600511	0.350257	-2.328911
H	1.982915	0.314021	2.676849	H	2.332840	-0.458608	-2.415718
H	0.622911	-0.702826	3.114599	H	1.344206	0.657419	-3.344913
C	-1.368698	1.601869	-0.719274	C	-1.281637	0.284190	1.131377
H	-1.043722	2.637113	-0.589994	H	-1.136665	1.101948	0.422337
H	-2.090097	1.593951	-1.536808	H	-2.361533	0.093787	1.146738
C	-2.001298	1.056597	0.579061	C	-0.553706	-0.980635	0.636008
H	-2.091119	1.844329	1.330500	H	-1.233922	-1.509028	-0.039034
H	-3.004687	0.673361	0.390445	H	1.631937	2.422379	-1.709939
exo-2				meso-8			
scf done: -466.897416				scf done: -466.829389			
C	0.246584	0.448050	1.332544	C	-3.162248	-0.429522	0.223937
C	2.686327	-0.797529	0.215613	C	-0.424052	-0.135073	-0.665254
H	0.109662	1.358082	1.919280	H	-4.159346	-0.547091	0.629785
H	3.626871	-1.207030	-0.137078	H	0.288464	-0.146838	-1.494892
C	-0.221243	0.574470	-1.150217	C	1.110747	1.448267	0.568729
H	0.162130	0.892423	-2.120416	C	0.408143	0.129814	0.638274
C	-0.521536	-0.939355	-1.161284	H	0.574499	2.349715	0.837186
H	0.403136	-1.489143	-1.342349	H	-0.304466	0.141529	1.467858
C	-1.182105	-0.114248	1.115022	C	1.434580	-0.977605	0.909460
H	-1.619408	-0.373679	2.080264	H	1.841571	-0.821546	1.911874
C	-1.149044	-1.345523	0.188831	H	0.949442	-1.954676	0.912856
H	-2.167922	-1.708839	0.048957	C	2.412866	1.535688	0.109800
C	2.156669	0.252559	-0.395088	H	2.871552	2.514656	0.022595
H	2.672185	0.689737	-1.245437	C	3.146611	0.424449	-0.250240
C	0.838848	0.874221	-0.044114	H	4.143892	0.542077	-0.655611
H	0.980642	1.957804	-0.043808	C	2.589419	-0.954217	-0.091947
C	2.024496	-1.454224	1.386922	H	2.254390	-1.330988	-1.067863
H	1.427220	-2.302836	1.039022	H	3.375949	-1.642303	0.229016
H	2.779044	-1.879898	2.052708	C	-1.126713	-1.453514	-0.595621
C	1.168769	-0.455780	2.167014	H	-0.590551	-2.354972	-0.864201
H	1.858872	0.191190	2.714789	C	-2.428695	-1.540830	-0.136279
H	0.574374	-0.978722	2.921360	H	-2.887464	-2.519736	-0.048856
C	-1.484213	1.298189	-0.775114	C	-1.450488	0.972354	-0.936396
H	-2.884927	1.362801	0.829873	H	-0.965323	1.949413	-0.939957
H	-1.932457	2.041275	-1.422772	H	-1.857635	0.816203	-1.938733
C	-1.981609	0.944073	0.404094	C	-2.605161	0.949131	0.065212
H	-1.197446	-1.164934	-1.986488	H	-3.391807	1.637040	-0.255854
H	-0.591063	-2.155995	0.657623	H	-2.270072	1.326208	1.040987
rac-8				syn-3			
scf done: -466.828436				scf done: -466.879118			
C	0.459509	3.167236	0.095575	C	2.479437	-4.244436	0.022341
C	-0.618747	0.477766	0.071227	C	3.979184	-2.450293	1.026224
H	0.923752	4.145185	0.119244	C	2.585883	-2.967713	0.889023

H	-1.509063	-0.157059	0.111530	C	3.548933	-4.443972	-1.041493
C	0.618747	-0.477766	0.071227	C	4.969531	-4.068778	-0.608606
H	1.509063	0.157059	0.111530	C	5.022903	-2.921462	0.354883
C	0.594057	-1.345975	1.294767	H	3.583565	-4.590696	5.075884
H	0.963272	-0.943840	2.229415	H	4.393117	-6.467377	3.757150
C	-0.459509	-3.167236	0.095575	H	4.127037	-1.629771	1.721688
H	-0.923752	-4.145185	0.119244	H	1.926084	-2.146213	0.598960
C	0.049942	-2.615001	1.254528	H	2.671017	-6.719616	3.585519
H	0.010886	-3.195389	2.169932	H	3.276079	-3.810607	-1.890618
C	-0.594057	1.345975	1.294767	H	3.528261	-5.473190	-1.408862
H	-0.963272	0.943840	2.229415	H	5.565826	-3.825652	-1.492464
C	-0.049942	2.615001	1.254528	H	5.471832	-4.928924	-0.154236
H	-0.010886	3.195389	2.169932	H	3.767646	-6.756208	1.340974
C	-0.711594	1.368641	-1.173799	H	5.998757	-2.476964	0.522848
H	-0.692682	0.773098	-2.087094	C	2.367423	-5.107511	1.312119
H	-1.682141	1.871440	-1.149794	C	2.611966	-3.689232	3.460038
C	0.384911	2.432430	-1.204375	C	2.039518	-3.811163	2.090663
H	0.205074	3.133209	-2.023842	C	3.607985	-5.805381	1.854761
H	1.355584	1.967348	-1.424802	C	3.480367	-6.014095	3.364092
C	0.711594	-1.368641	-1.173799	C	3.227184	-4.702615	4.057473
H	0.692682	-0.773098	-2.087094	H	1.546617	-5.828248	1.286916
H	1.682141	-1.871440	-1.149794	H	4.492232	-5.191020	1.691328
C	-0.384911	-2.432430	-1.204375	H	2.479009	-2.748478	3.985392
H	-1.355584	-1.967348	-1.424802	H	0.957034	-3.666521	2.142805
H	-0.205074	-3.133209	-2.023842	H	1.504177	-4.262643	-0.468497
threo-4				TS_meso-8_exo-2			
scf done: -466.883203				scf done: -466.816232			
C	2.719385	0.885487	0.238452	C	-1.316506	0.192706	1.584009
C	0.811128	-1.173216	-0.430027	C	-1.738119	1.221052	0.574432
H	3.315905	1.692771	0.645462	C	-2.369383	0.843115	-0.627952
H	0.700694	-2.088483	-1.019779	C	-2.056673	-0.350699	-1.200833
C	-0.544129	-1.027413	0.324005	C	-1.017744	-1.172365	-0.515661
H	-0.601124	-1.925539	0.951237	C	-1.372596	-1.211633	0.981935
C	-1.694434	-1.102609	-0.640668	H	-3.003479	1.554969	-1.145710
H	-1.762342	-2.008632	-1.236795	H	-1.805720	2.249740	0.907981
C	-2.604231	1.139335	-0.040504	H	-0.314058	0.424924	1.955902
H	-3.372369	1.101374	0.741328	H	-1.976772	0.250492	2.456734
C	-2.612119	-0.156971	-0.796028	H	-2.479075	-0.658821	-2.148471
H	-3.424746	-0.319208	-1.497050	H	-1.011953	-2.192325	-0.905349
C	1.932980	-1.395249	0.548309	H	-2.387097	-1.606085	1.065262
H	1.978704	-2.357375	1.047347	H	-0.728056	-1.896949	1.532026
C	2.803160	-0.435773	0.859643	C	0.503661	0.913569	-0.684025
H	3.574513	-0.615059	1.599120	C	0.425859	-0.580900	-0.777418
C	1.147135	-0.044094	-1.418299	C	1.470282	-1.260774	0.120030
H	0.231310	0.319936	-1.888490	H	1.150512	-1.201744	1.160855
H	1.755071	-0.458649	-2.232187	H	1.530261	-2.322397	-0.132207
C	1.925198	1.086850	-0.810397	C	2.844192	-0.606280	0.042055
H	1.868486	2.062294	-1.279661	H	3.513782	-1.063866	0.773679
H	-2.898599	1.954916	-0.706296	H	3.300867	-0.786043	-0.940318
C	-0.673830	0.182601	1.261605	C	1.625259	1.547113	-0.107560

H	0.291051	0.404709	1.718528
H	-1.349543	-0.092967	2.077297
C	-1.239245	1.423703	0.577245
H	-0.553137	1.763233	-0.200781
H	-1.315098	2.240309	1.298182

H	1.610074	2.628588	-0.022372
C	2.738394	0.868989	0.271030
H	3.591460	1.402139	0.673157
H	0.652177	-0.850275	-1.816962
H	-0.117495	1.493564	-1.345777

TS_meso-8_syn-3

scf done: -466.814947

C	-1.455656	-2.800496	-0.952015
C	-0.536282	-0.076436	-0.551513
H	-1.759847	-3.835854	-1.051902
H	-0.527323	0.923936	-0.992442
C	1.740745	-0.574764	0.082300
C	0.450545	-0.028186	0.653660
H	2.301339	0.065186	-0.587187
H	0.598762	1.025508	0.910344
C	0.085676	-0.722749	1.977024
H	0.655614	-0.213241	2.758158
H	-0.966483	-0.556884	2.216924
C	2.355652	-1.729371	0.581589
H	3.321726	-2.010446	0.176671
C	1.766505	-2.533632	1.508790
H	2.261292	-3.441355	1.831887
C	0.423907	-2.212557	2.077066
H	-0.323934	-2.820727	1.562502
H	0.377618	-2.516748	3.126497
C	0.088070	-1.027613	-1.548989
H	0.735480	-0.635815	-2.320793
C	-0.383571	-2.342745	-1.657005
H	0.123342	-3.019056	-2.336717
C	-2.003950	-0.428183	-0.290942
H	-2.395197	0.157931	0.543994
H	-2.566829	-0.117844	-1.175441
C	-2.271356	-1.918623	-0.063418
H	-3.333635	-2.123076	-0.227707
H	-2.094608	-2.185553	0.984584

TS_rac-8_anti-3

scf done: -466.822811

C	0.204455	2.936654	0.724551
C	-0.621306	0.459437	-0.542246
H	0.530773	3.857554	1.192424
H	-1.475560	-0.094962	-0.942334
C	0.621306	-0.459437	-0.542246
H	1.475560	0.094962	-0.942334
C	0.885956	-0.779847	0.913879
H	1.550630	-0.139012	1.475954
C	-0.204455	-2.936654	0.724551
H	-0.530773	-3.857554	1.192424
C	0.486474	-2.011982	1.451060
H	0.716185	-2.221386	2.490164
C	-0.885956	0.779847	0.913879
H	-1.550630	0.139012	1.475954
C	-0.486474	2.011982	1.451060
H	-0.716185	2.221386	2.490164
C	-0.444549	1.732021	-1.371306
H	-0.105013	1.472966	-2.377771
H	-1.415902	2.222707	-1.475775
C	0.529447	2.714038	-0.718430
H	0.520885	3.663659	-1.258773
H	1.557112	2.336137	-0.808649
C	0.444549	-1.732021	-1.371306
H	0.105013	-1.472966	-2.377771
H	1.415902	-2.222707	-1.475775
C	-0.529447	-2.714038	-0.718430
H	-1.557112	-2.336137	-0.808649
H	-0.520885	-3.663659	-1.258773

TS_rac-8_endo-2

scf done: -466.821312

C	2.273469	1.069172	0.172494
C	0.390481	-0.910393	-0.721117
H	2.921191	1.837589	0.578378
H	0.053954	-1.584737	-1.513802
C	-0.645723	-1.156217	0.462404
H	-0.393656	-2.134375	0.877359
C	-0.577430	-0.107494	1.511251
H	-0.023562	-0.269122	2.425889
C	-1.794912	1.283815	0.000413
H	-2.039069	2.286634	-0.330274
C	-1.128180	1.105595	1.216061
H	-0.951733	1.959091	1.860603
C	0.400904	0.483185	-1.252430

TS-5

scf done: -466.787220

C	0.290123	0.694091	1.544034
C	2.436200	-0.688130	0.198779
H	-0.128393	1.401749	2.249289
H	3.268258	-1.181560	-0.290368
C	-1.151979	-1.360591	-1.008943
H	-0.978605	-2.326243	-1.470318
C	-1.362367	-1.275575	0.299493
H	-1.338070	-2.167905	0.913729
C	-1.992534	1.073847	0.140921
H	-2.402853	1.978963	0.574359
C	-1.658503	0.010552	0.961128
H	-2.080520	-0.044107	1.957525
C	2.061335	0.527343	-0.189259

H	-0.190615	0.733918	-2.120705	H	2.601202	1.030351	-0.984069
C	1.349122	1.398257	-0.771098	C	0.922108	1.241541	0.410190
H	1.307581	2.420130	-1.132152	H	0.973050	2.326250	0.355483
C	1.811349	-1.336282	-0.321273	C	1.704388	-1.437464	1.270314
H	1.789514	-2.331731	0.128692	H	1.024332	-2.147469	0.792261
H	2.403564	-1.415421	-1.236764	H	2.404278	-2.038227	1.856214
C	2.502667	-0.344888	0.612898	C	0.923544	-0.511102	2.210112
H	3.577761	-0.549496	0.627395	H	1.620502	-0.108552	2.955506
H	2.168743	-0.481274	1.645608	H	0.185755	-1.088133	2.772074
C	-2.071757	-1.208301	-0.103493	C	-1.170030	-0.157280	-1.902898
H	-2.150612	-1.992121	-0.860479	H	-0.239616	-0.110769	-2.475208
H	-2.746930	-1.479935	0.710211	H	-1.971477	-0.262077	-2.645407
C	-2.489921	0.145432	-0.687921	C	-1.350795	1.130686	-1.107723
H	-2.303964	0.183277	-1.764881	H	-0.140764	1.268426	-0.572101
H	-3.572779	0.273836	-0.579796	H	-1.528295	2.013278	-1.716979
TS-6				TS'-5			
scf done: -466.793979				scf done: -466.791921			
C	-0.941010	-0.977173	1.029971	C	0.249340	0.604360	1.703145
C	-1.965625	1.247674	-0.448169	C	2.078369	-0.652920	-0.101589
H	-0.935067	-1.722719	1.825325	H	-0.089434	1.198743	2.543826
H	-2.372121	2.092593	-0.991558	H	2.790967	-1.114337	-0.775524
C	1.620099	-1.410794	0.077724	C	-1.579631	-0.140824	-0.157159
H	1.874039	-2.449860	0.261004	H	-1.300933	-1.130268	-0.497066
C	1.978177	-0.461500	1.037022	C	-1.708224	0.121115	1.199780
H	2.268717	-0.795609	2.026697	H	-1.853093	-0.708659	1.881187
C	1.386575	1.320304	-0.517572	C	-2.567137	2.367910	0.693885
H	1.237534	2.380050	-0.680759	H	-3.079392	3.275038	0.993979
C	1.640229	0.854911	0.838596	C	-2.362236	1.395856	1.574733
H	1.823643	1.588719	1.611723	H	-2.675202	1.518037	2.605553
C	-1.378112	1.435946	0.740233	C	1.847528	0.657808	-0.175073
H	-1.313352	2.435111	1.156145	H	2.375000	1.255628	-0.910281
C	-0.774624	0.353072	1.476828	C	0.827373	1.329108	0.640595
H	-0.445183	0.542369	2.487636	H	0.876682	2.409073	0.719444
C	-2.060727	-0.112921	-1.071785	C	1.391109	-1.528447	0.910514
H	-1.232293	-0.240506	-1.778900	H	0.563518	-2.069198	0.441477
H	-2.975651	-0.197188	-1.662466	H	2.080406	-2.298421	1.265672
C	-2.019331	-1.216786	-0.017316	C	0.892229	-0.708393	2.098351
H	-2.986933	-1.253184	0.495235	H	1.753532	-0.458714	2.730045
H	-1.880661	-2.188058	-0.496454	H	0.221526	-1.306133	2.718226
C	1.510101	-1.004867	-1.374514	C	-1.343665	0.972122	-0.984405
H	0.675827	-1.532102	-1.853304	H	-0.216728	1.264920	-0.406474
H	2.402080	-1.333283	-1.926095	H	-1.128146	0.771257	-2.030489
C	1.335802	0.475313	-1.545826	C	-2.139278	2.249585	-0.738535
H	1.181694	0.848859	-2.551610	H	-1.533858	3.117370	-1.016739
H	0.183496	-1.310030	0.506740	H	-3.020770	2.282240	-1.391044
TS'-6				TS-endo-2			
scf done: -466.795048				scf done: -466.809179			
C	0.726535	0.808706	-0.906969	C	-0.410727	0.580707	-1.214336
C	2.512451	-1.121034	0.295632	C	-0.443641	-0.785486	-0.951182
H	0.203095	1.546204	-1.497231	H	0.177077	0.976945	-2.028025

H	1.958782	-1.479239	1.171580	H	0.094058	-1.423163	-1.644846
C	-0.722878	0.148132	1.411475	C	-1.757870	-1.374325	-0.470707
H	0.054600	0.164441	2.166212	H	-2.400035	-1.466878	-1.353738
C	-0.983038	1.287858	0.687118	H	-1.617243	-2.386525	-0.087718
H	-0.590889	2.242280	1.011499	C	-1.389837	1.431157	-0.575758
C	-2.790691	0.171665	-0.520025	H	-1.335473	2.498401	-0.758303
H	-3.635355	0.204061	-1.198555	C	-2.291160	0.957474	0.292014
C	-2.096958	1.278857	-0.260820	H	-2.949352	1.646913	0.809250
H	-2.356285	2.208128	-0.755199	C	-2.475315	-0.509067	0.564947
C	1.750330	-1.468478	-0.983667	H	-3.542387	-0.748325	0.558134
H	1.440695	-2.515231	-0.962550	H	-2.131850	-0.743994	1.575623
C	0.543320	-0.559886	-1.188306	C	2.429164	0.234776	-0.720608
H	-0.045463	-0.782681	-2.074456	C	1.647345	1.311117	-0.017934
C	2.729635	0.361710	0.415060	C	1.065069	1.110557	1.204217
H	3.572708	0.715263	0.997070	C	0.631648	-0.173806	1.551350
C	1.899128	1.234514	-0.161445	C	0.838801	-1.193933	0.633059
H	2.074554	2.299151	-0.054182	C	2.145775	-1.157539	-0.131869
C	-1.160113	-1.075331	0.895357	H	0.736583	1.960505	1.789029
H	-0.249862	-0.975738	-0.229494	H	1.797024	2.324656	-0.373641
H	-0.875502	-1.983235	1.419179	H	2.230954	0.262268	-1.795172
C	-2.468755	-1.140954	0.133537	H	3.494807	0.458162	-0.615889
H	-2.418309	-1.931100	-0.622065	H	-0.022753	-0.310267	2.401784
H	-3.290457	-1.432024	0.803042	H	0.474174	-2.185047	0.881157
H	2.422816	-1.352374	-1.840358	H	2.937299	-1.417655	0.575804
H	3.469623	-1.646631	0.312066	H	2.163847	-1.920215	-0.911902
TS-exo-2				TS-meso			
scf done: -466.806816				scf done: -466.807516			
C	-1.117477	0.631159	1.489729	C	-3.118830	-0.432302	0.247332
C	-1.492900	1.406172	0.261074	C	-0.504203	-0.164432	-0.850126
C	-2.313518	0.873124	-0.703567	H	-4.083761	-0.548950	0.726227
C	-2.291275	-0.505193	-0.928118	H	0.357036	-0.115956	-1.507732
C	-1.422929	-1.266287	-0.162964	C	1.103088	1.417337	0.681717
C	-1.284621	-0.887607	1.293464	C	0.504203	0.164432	0.850126
H	-2.791881	1.526904	-1.423749	H	0.542386	2.320926	0.884590
H	-1.363101	2.481322	0.313325	H	-0.357036	0.115956	1.507732
H	-0.101124	0.894014	1.791604	C	1.462962	-0.999844	0.987640
H	-1.762987	0.962630	2.307950	H	1.899696	-0.939899	1.990513
H	-2.742149	-0.913678	-1.824459	H	0.928195	-1.948593	0.935140
H	-1.332677	-2.325074	-0.382214	C	2.407563	1.521812	0.117492
H	-2.199823	-1.219513	1.791484	H	2.824596	2.508834	-0.046533
H	-0.469934	-1.439565	1.761269	C	3.118830	0.432302	-0.247332
C	0.530452	0.735575	-0.931303	H	4.083761	0.548950	-0.726227
C	0.461567	-0.657369	-0.933504	C	2.592220	-0.958547	-0.043492
C	1.514567	-1.424974	-0.158548	H	2.249090	-1.356941	-1.006753
H	1.136506	-2.397615	0.165008	H	3.401172	-1.622287	0.271158
H	2.321990	-1.638756	-0.869017	C	-1.103088	-1.417337	-0.681717
C	2.110504	-0.677183	1.041972	H	-0.542386	-2.320926	-0.884590
H	1.476480	-0.811478	1.925144	C	-2.407563	-1.521812	-0.117492
H	3.071714	-1.123142	1.307429	H	-2.824596	-2.508834	0.046533
C	1.523326	1.422412	-0.125007	C	-1.462962	0.999844	-0.987640

H	1.641533	2.489814	-0.273526
C	2.272163	0.790740	0.782103
H	2.994852	1.340906	1.373193
H	0.200651	-1.122403	-1.874173
H	0.119794	1.278603	-1.768221

H	-0.928195	1.948593	-0.935140
H	-1.899696	0.939899	-1.990513
C	-2.592220	0.958547	0.043492
H	-3.401172	1.622287	-0.271158
H	-2.249090	1.356941	1.006753

TS-rac
scf done: -466.801613

C	0.438518	3.178150	-0.083811
C	-0.806943	0.631402	0.284646
H	0.939819	4.130385	-0.207222
H	-1.538041	-0.152793	0.444670
C	0.806943	-0.631402	0.284646
H	1.538041	0.152793	0.444670
C	0.578856	-1.510314	1.355638
H	0.784838	-1.187242	2.368494
C	-0.438518	-3.178150	-0.083811
H	-0.939819	-4.130385	-0.207222
C	-0.025715	-2.777016	1.142026
H	-0.182307	-3.426756	1.995461
C	-0.578856	1.510314	1.355638
H	-0.784838	1.187242	2.368494
C	0.025715	2.777016	1.142026
H	0.182307	3.426756	1.995461
C	-0.867039	1.290978	-1.077537
H	-0.883142	0.557052	-1.882941
H	-1.825052	1.822189	-1.130670
C	0.264182	2.296637	-1.285189
H	0.067687	2.898777	-2.175067
H	1.206265	1.769226	-1.485919
C	0.867039	-1.290978	-1.077537
H	0.883142	-0.557052	-1.882941
H	1.825052	-1.822189	-1.130670
C	-0.264182	-2.296637	-1.285189
H	-1.206265	-1.769226	-1.485919
H	-0.067687	-2.898777	-2.175067

TS-retro-ene
scf done: -466.815315

C	-1.186691	2.181411	1.255581
C	-0.640266	-0.563571	0.769034
H	-1.416671	3.226221	1.422690
H	-0.836686	-1.615953	0.919152
C	0.799446	-1.076613	-0.742842
H	0.259899	-1.921626	-1.157367
C	1.831538	-1.337636	0.147239
H	1.871657	-2.306864	0.634256
C	2.708165	1.004883	-0.056743
H	3.619082	0.888760	-0.653952
C	2.503000	-0.271490	0.728322
H	3.252829	-0.489118	1.483596
C	-1.620994	0.155255	0.009056
H	-2.187631	-0.385991	-0.739966
C	-1.845148	1.477642	0.199505
H	-2.573814	1.996970	-0.410536
C	0.038647	0.146050	1.847606
H	1.300189	0.110677	1.511093
H	0.199208	-0.432543	2.756949
C	-0.329118	1.532579	2.072140
H	0.119671	2.048379	2.913009
H	2.894534	1.839347	0.624101
C	0.918493	0.119332	-1.654615
H	-0.057791	0.384528	-2.065452
H	1.540113	-0.180845	-2.505556
C	1.521016	1.341390	-0.962043
H	0.745933	1.816652	-0.363650
H	1.825458	2.076420	-1.709859

TS-threo-4
scf done: -466.801963

C	1.741500	1.339935	0.183141
C	0.784040	-1.346914	0.248648
H	2.072878	2.367792	0.095506
H	0.870290	-2.405357	0.493458
C	-0.688606	-1.284761	-0.552263
H	-0.725137	-2.256051	-1.045134
C	-1.759558	-1.147818	0.424371
H	-2.083774	-2.018151	0.983312
C	-1.800247	1.255064	0.135879
H	-2.186001	2.213270	0.462741
C	-2.245407	0.090778	0.760622
H	-2.960772	0.177436	1.570894
C	1.841099	-0.926527	-0.659516

H	2.210319	-1.621427	-1.404999	
C	2.252341	0.382033	-0.692385	
H	2.954137	0.698490	-1.456071	
C	0.845685	-0.546051	1.552936	
H	-0.013878	-0.788538	2.180350	
H	1.739039	-0.879834	2.088543	
C	0.934454	0.964076	1.345198	
H	1.277673	1.470521	2.250196	
H	-0.118110	1.356220	1.206153	
C	-0.797200	-0.197224	-1.625315	
H	0.070077	-0.237142	-2.286559	
H	-1.675776	-0.434209	-2.232475	
C	-0.960702	1.209504	-1.056625	
H	0.079113	1.591447	-0.792825	
H	-1.299928	1.914880	-1.818317	
